# *Pediococcus pentosaceus* LI05 alleviates DSS‐induced colitis by modulating immunological profiles, the gut microbiota and short‐chain fatty acid levels in a mouse model

**DOI:** 10.1111/1751-7915.13583

**Published:** 2020-05-03

**Authors:** Xiaoyuan Bian, Liya Yang, Wenrui Wu, Longxian Lv, Xianwan Jiang, Qing Wang, Jingjing Wu, Yating Li, Jianzhong Ye, Daiqiong Fang, Ding Shi, Kaicen Wang, Qiangqiang Wang, Yanmeng Lu, Jiaojiao Xie, Jiafeng Xia, Lanjuan Li

**Affiliations:** ^1^ State Key Laboratory for Diagnosis and Treatment of Infectious Disease National Clinical Research Center for Infectious Diseases The First Affiliated Hospital Zhejiang University Hangzhou China; ^2^ Collaborative Innovation Center for Diagnosis and Treatment of Infectious Diseases Hangzhou China

## Abstract

The gut microbiota is considered a key factor in pathogenesis and progression of inflammatory bowel disease (IBD). The bacterium *Pediococcus pentosaceus* LI05 alleviated host inflammation by maintaining the gut epithelial integrity, modulating the host immunity, gut microbiota and metabolism, but its effect on IBD remains unclear. The present study aimed to investigate the role and mechanisms of *P. pentosaceus* LI05. Mice were administered *P. pentosaceus* LI05 or phosphate‐buffered saline once daily by oral gavage for 14 days, and colitis was induced by providing mice 2% DSS‐containing drinking water for 7 days. *P. pentosaceus* LI05 ameliorated colitis in mice and reduced the body weight loss, disease activity index (DAI) scores, colon length shortening, intestinal permeability and the proinflammatory cytokine levels. Furthermore, a significantly altered gut microbiota composition with increased diversity and short‐chain fatty acid (SCFA) production was observed in mice treated with *P. pentosaceus* LI05. Several genera, including *Akkermansia* and *Faecalibacterium,* were differentially enriched in the *P. pentosaceus* LI05‐treated mice and were negatively correlated with colitis indices and positively correlated with gut barrier markers and SCFA levels. The *P. pentosaceus* LI05 treatment alleviated intestinal inflammation by maintaining the intestinal epithelial integrity and modulating the immunological profiles, gut microbiome and metabolite composition. Based on our findings, *P. pentosaceus* LI05 might be applied as potential preparation to ameliorate colitis.

## Graphical abstract

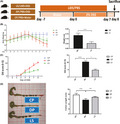

## Introduction

Inflammatory bowel disease (IBD), consisting of ulcerative colitis (UC) and Crohn’s disease (CD), is a group of disorders causing chronic inflammation of the gastrointestinal tract (Wlodarska *et al.*, 2015; Franzosa *et al.*, 2019). Multiple factors, such as genetics, host immunity and the intestinal microbiome, participate in the initial progression of the diseases (Liu *et al.*, 2019). The gut microbiota comprises a wide range of gut microbes that closely interact with the host to enhance the epithelial barrier function and regulate the host immunity and metabolism (Vemuri *et al.*, 2017, 2018). In addition, the composition of the intestinal microbiota in patients with IBD markedly differs from healthy people (Blander *et al.*, 2017; Lane *et al.*, 2017). Likewise, several animal experiments observed major changes in the structure of the gut microbiota in response to chemically induced colitis (Souza *et al.*, 2015; Llewellyn *et al.*, 2018). Despite the strong correlation between an altered intestinal microbiota structure and diseases, certain underlying mechanisms remain unknown and potential targets for intervention must be identified.

Probiotics are living microbes that exert a demonstrated beneficial effect on human health if administered at effective doses. Probiotics have been found to protect against colitis in both experimental models and human studies (Bibiloni *et al.*, 2005; Souza *et al.*, 2015; Ahl *et al.*, 2016). Genera such as *Lactobacillus*, *Faecalibacterium* and *Akkermansia* exert anti‐inflammatory effects on the intestinal mucosa of patients with IBD (Tamboli *et al.*, 2004; Sokol *et al.*, 2009; Bian *et al.*, 2019). Specifically, *Faecalibacterium prausnitzii* exerts significant beneficial effects on colitis by producing the short‐chain fatty acid (SCFA) butyrate (Qiu *et al.*, 2013; Rossi *et al.*, 2015). Additionally, *Lactobacillus rhamnosus* Gorbach‐Goldin (LGG) and *Akkermansia muciniphila* strengthen the epithelial barrier function and protect against epithelial inflammation (Chen *et al.*, 2016; Wu *et al.*, 2017; Mantegazza *et al.*, 2018).

*Pediococcus pentosaceus,* a member of the family *Lactobacillaceae*, is a species that has been shown to ameliorate inflammation (Lv *et al.*, 2014a). In addition, several species of *P. pentosaceus* have been reported to alleviate encephalopathy, acute liver failure, obesity and fatty liver (Bengmark *et al.*, 2011; Zhao *et al.*, 2012; Shi *et al.*, 2017). Our newly isolated probiotic *P. pentosaceus* LI05 has been shown to protect the host mucosa by strengthening the epithelial barrier function and regulating the host immunity and gut microbiota in mouse models of *Clostridium difficile* infection and CCl4‐induced cirrhosis (Shi *et al.*, 2017; Xu *et al.*, 2018). Based on these findings, *P. pentosaceus* LI05 may exert a beneficial and effect on preventing intestinal inflammation. However, its precise role in colitis and the potential mechanisms underlying these beneficial effects remain unknown.

In the present study, a DSS‐induced colitis model was established to explore the dynamic role of the intestinal microbiome. The combination of measurements of cytokine levels, the gut barrier function, 16S rRNA gene sequencing, and gut metabolite and SCFA levels was used to understand the protective role *P. pentosaceus* LI05 and identify the potential mechanisms involved.

## Results

### *P. pentosaceus* LI05 ameliorated DSS‐induced clinical features and intestinal injury

**Fig. 1:**
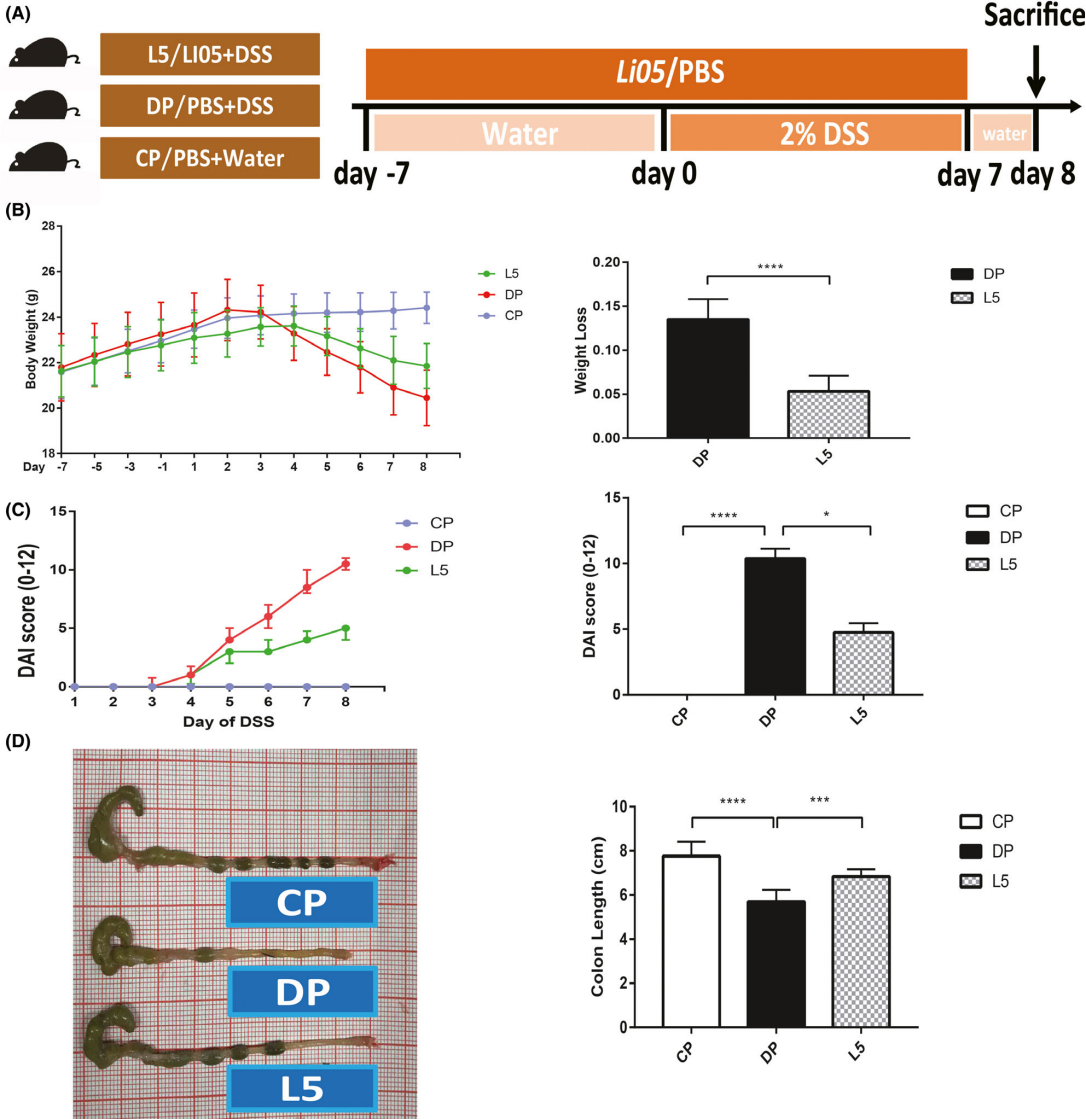
*P. pentosaceus* LI05 ameliorated DSS‐induced clinical symptoms and intestinal injury in mice. A. Schematic depicting the design of the animal experiment. B. The body weights of mice in the three groups recorded from day −7 to day 8 are plotted and presented as mean (SD; left panel). The bar chart represents the weight loss relative to the base weight on day 0 (right panel). C. The disease activity index (DAI) scores after DSS administration (left panel) and on day 8 (right panel) are shown for the three groups. D. Representative images of the colon in mice from the three groups (left panel) and the colon length on day 8 (right panel). *P < 0.05, ***P < 0.001 and ****P < 0.0001 according to unpaired t tests with Welch’s correction, Kruskal–Wallis tests and post hoc one‐way ANOVA.

A DSS‐induced colitis model was successfully established to evaluate the effect of *P. pentosaceus* LI05 (LI05) on mice. Treatment with LI05 significantly reduced the weight loss (Fig. 1B), DAI score (Fig. 1C) and colon length shortening (Fig. 1D) induced by DSS consumption.

**Fig. 2:**
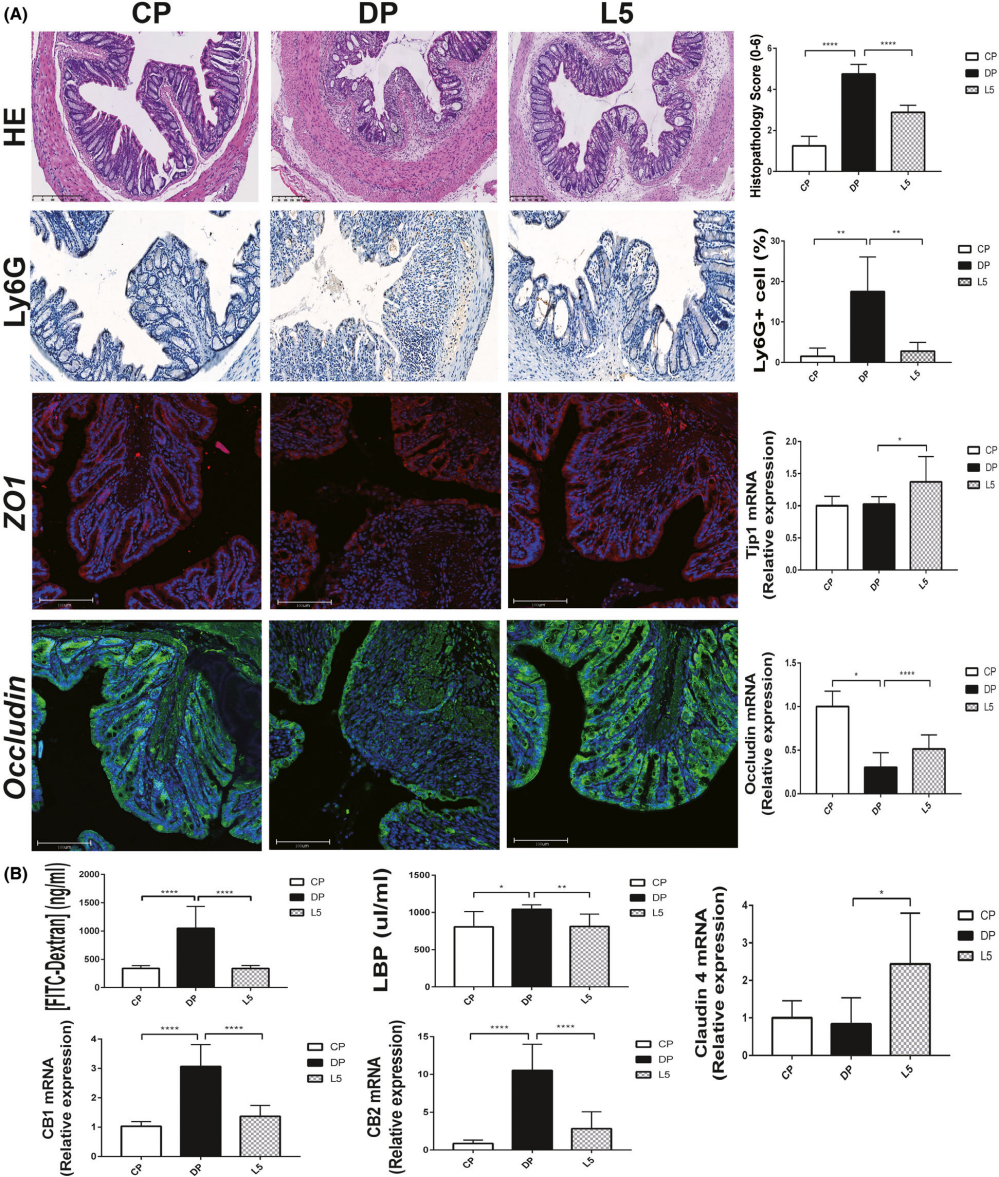
*P. pentosaceus* LI05 ameliorated colon epithelial damage. A. Representative images of H&E staining, Ly6G immunohistochemical staining, ZO1 and Occludin immunofluorescence staining among three groups (left panel). Histopathology scores, the percentage of Ly6G‐positive cells, and colon expression of ZO1 (Tjp1) and Occludin in the three groups (right panel). B. The bar chart presents the plasma concentration of FITC–dextran, serum LBP levels and colon expression of CB1, CB2 and Claudin 4 among three groups. All data are presented as means ± SEM. *P < 0.05, **P < 0.01, ****P < 0.0001 according to the post hoc one‐way ANOVA.

Gastric mucosal barrier destruction is the initiating event of all known chemically induced colitis models. The main finding in our model was marked inflammatory cell infiltration (predominantly neutrophils) and a barely intact mucosal architecture with crypt abscesses and ulceration (Fig. 2A). However, mucosal damage was ameliorated in mice treated with LI05. A preserved mucosal architecture with minimal goblet cell loss and mild/moderate inflammatory infiltration was observed in the L5 group. In addition, the histopathology scores (*P* < 0.0001) and infiltration of neutrophils (Ly6G‐positive cells, *P* < 0.01) were markedly decreased in the L5 group compared to the DP group (Fig. 2A).

As mentioned above, the administration of DSS caused severe intestinal mucosal damage, and the LI05 treatment ameliorated the symptoms. We hypothesized that LI05 might exert its protective effects by restoring the intestinal mucosal damage and enhancing the mucosal barrier function. We assessed Occludin*,* ZO1 and Claudin 4 expression using immunofluorescence staining and quantitative PCR to test this hypothesis. As presented in Figure 2A, colon tissue of L5 group showed increased ZO1 fluorescence intensities and stabilized mucosal integrity. The expression of tight junction proteins (Tjps) mRNA in the colon tissue was upregulated in L5 group by contrast with DP group (Occludin, *P* < 0.0001; ZO1/Tjp1, *P* < 0.05; Claudin 4, *P* < 0.05; Fig. 2A and B).

Intestinal permeability was assessed by measuring FITC–dextran, LBP and cannabinoid receptor (CB) levels to further verify this hypothesis. The DP group presented increased FITC–dextran (*P* < 0.0001), CB1 (*P* < 0.0001), CB2 (*P* < 0.0001) and serum LBP (*P* < 0.05) levels, whereas the LI05 treatment reversed this trend (Fig. 2B; *P* < 0.0001, *P* < 0.0001, *P* < 0.0001 and *P* < 0.01, respectively, Fig. 2B).

### *P. pentosaceus* LI05 ameliorated DSS‐induced colitis, displaying anti‐inflammatory properties

**Fig. 3:**
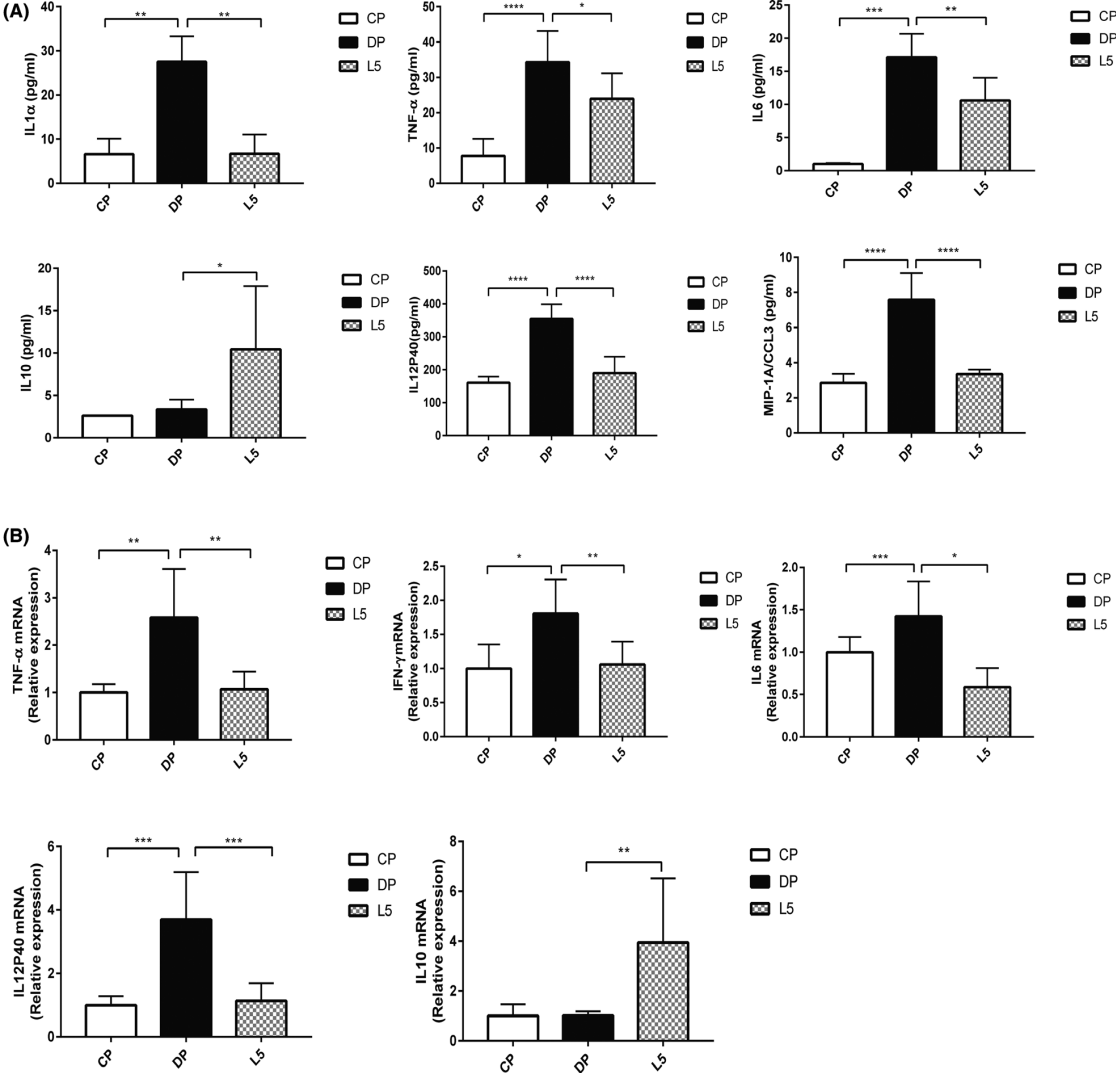
*P. pentosaceus* LI05 ameliorated DSS‐induced colitis by exerting anti‐inflammatory effects. A. The serum levels of the cytokines IL1α, TNF‐α, IL6, IL10, IL12P40 and MIP‐1A in the three groups. B. Colon expression of TNF‐α, IFN‐γ, IL6, IL12P40 and IL10 in the three groups. All data are presented as means ± SEM. *P < 0.05, **P < 0.01, ***P < 0.001 and ****P < 0.0001 according to the post hoc one‐way ANOVA.

The restoration of intestinal permeability might be correlated with a decrease in the immunological response (Genser *et al.*, 2016). Therefore, the protective effects of *P. pentosaceus* LI05 on intestinal inflammation were explored by determining the serum and colon tissue cytokine levels. Consistent with the results of a previous experiment (Bian *et al.*, 2019), increased levels of the cytokines IL1α, TNF‐α, IL6, IL12P40 and MIP‐1A were observed in mice with DSS‐induced colitis. Notably, the administration of LI05 significantly decreased the levels of these cytokines. (*P* < 0.01, *P* < 0.05, *P* < 0.01, *P* < 0.0001 and *P* < 0.0001, respectively, Fig. 3A). When compared to CP group, the expression of TNF‐α, IFN‐γ, IL6 and IL12P40 in colon tissue was markedly increased in mice with DSS administration (*P* < 0.01, *P* < 0.05, *P* < 0.001 and *P* < 0.001; Fig. 3B). In the LI05 group, these expression levels were significantly downregulated and practically returned to the normal level (*P* < 0.01, *P* < 0.01, *P* < 0.05 and *P* < 0.001 respectively). Furthermore, the immunomodulatory cytokine IL10 levels upregulated in both the serum and colon after *P. pentosaceus* LI05 treatment (*P* < 0.05, *P* < 0.01; Fig. 3A and B).

### *P. pentosaceus* LI05 ameliorated gut microbiome diversity upon DSS administration

**Fig. 4:**
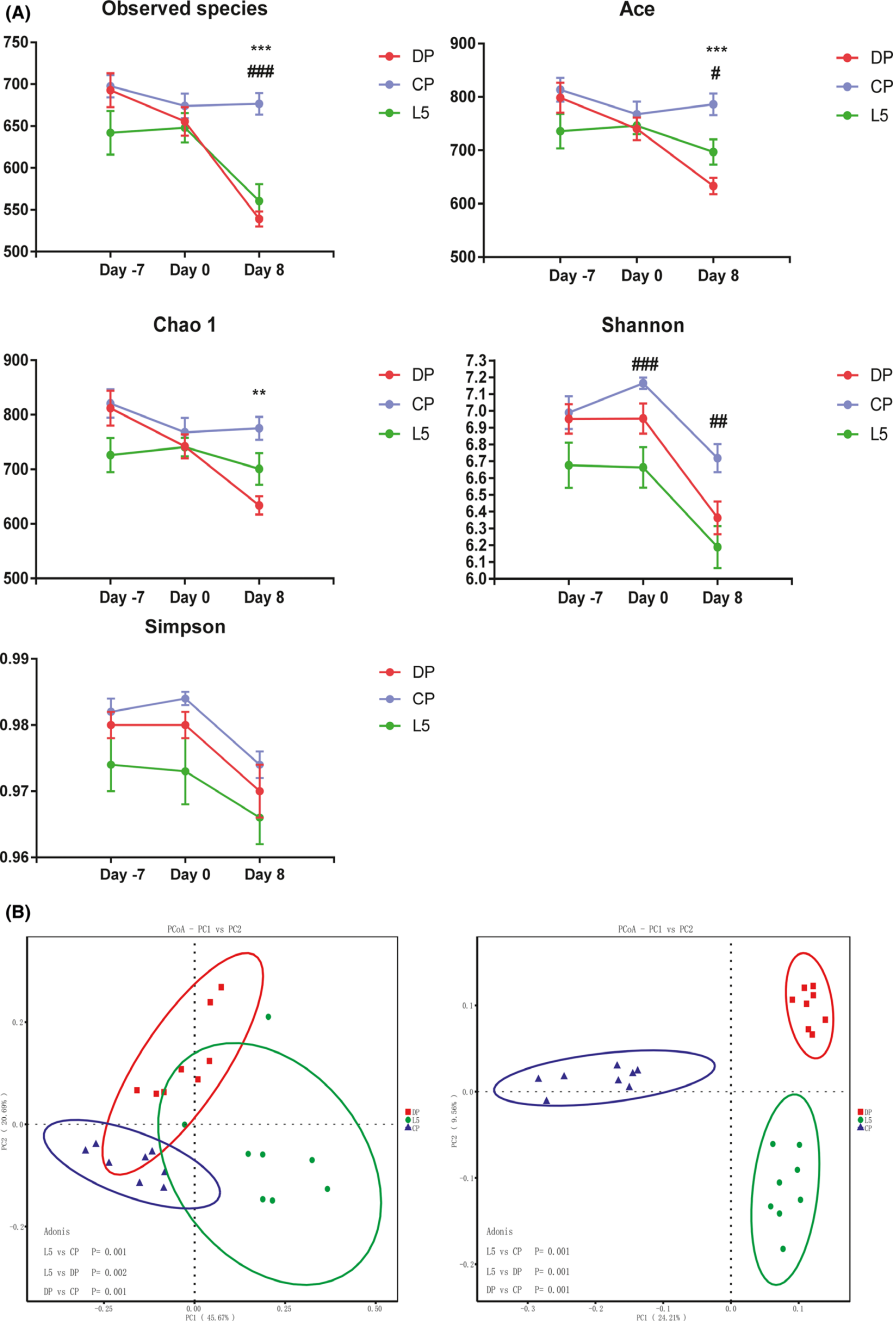
*P. pentosaceus* LI05 ameliorated gut microbiome diversity induced by DSS administration. A. Richness and diversity of the faecal microbiota in the three groups. Abbreviation: *CP group compared to the DP group. ^#^CP group compared to the L5 group. All data are presented as means ± SEM. B. PCoA plot based on the weighted (left panel) and unweighted (right panel) UniFrac distances among the three groups (day 8). Each point represents a sample.

We investigated the effects of *P. pentosaceus* LI05 intake on the gut microbiota composition using a 16S rRNA sequencing analysis to further explore the processes mediating the protective effects of *P. pentosaceus* LI05. Seventy‐two faecal samples were collected from the three groups (*n* = 8 each) at three different time points (day −7, day 0 and day 8), and the identified OTU numbers are presented in Table 1. During the experiment, the OTU numbers decreased as the time of DSS administration increased in the DP (*P* < 0.001, day 8 vs. day 0; *P* < 0.001, day 8 vs. day −7) and L5 (*P* < 0.05, day 8 vs. day 0; *P* < 0.05, day 8 vs. day −7) groups. At the end of the experiment (day 8), markedly lower OTU numbers were obtained from the DP and L5 groups (*P* < 0.001 and *P* < 0.001 respectively) than from the CP group after DSS exposure. However, a significant difference in OTU numbers was not observed between the DP and L5 groups. Throughout the experiment, the species diversity and community richness of the gut microbiome, as calculated by the observed species, Ace, Chao1, Shannon and Simpson indices, did not change significantly in the CP group (Fig. 4A). Additionally, the observed species, Ace and Chao1 indices exhibited marked reductions after DSS administration (day 8, DP vs. CP: *P* < 0.001, *P P* < 0.001 and *P* < 0.01, respectively, Fig. 4A). No significant differences in these indices were observed between the DP group and L5 group, although the L5 group showed increasing trends. The species accumulation box plot reflects the species richness and sequencing depth of the analysis (Figure S1). Next, both weighted and unweighted UniFrac PCoAs, reflecting alterations in the species complexity and composition, respectively, were calculated and are presented in Figure 4B, Figure S2 and Table S2. Differences were not observed among the three groups at baseline (day −7, Fig. S2). After 7 days of LI05 administration, the L5 group was significantly separated from the CP group (day 0, *P* = 0.012, Fig. S2). At the end of the experiment (day 8), the CP group and the DP group formed two distinct clusters (*P* = 0.001, A > 0, Table S2). Likewise, the L5 group was clearly separated from the DP and CP groups (L5 vs. DP, *P* = 0.003, A > 0; L5 vs. CP, *P* = 0.001, A > 0, Table S2), indicating that *P. pentosaceus* LI05 exerts an important effect on regulating the composition of the gut microbiota.

**Table 1:** OTU numbers of faecal microbiota among groups in different time.

Group	OTUs		
	Day −7	Day 0	Day 8
CP	776.00 ± 15.17	749.88 ± 18.11	755.75 ± 17.66
DP	767.88 ± 20.76	726.63 ± 18.33	607.38 ± 7.64^***###^^^^
L5	743.88 ± 23.79	727.13 ± 15.34	651.75 ± 19.75^*#^^^^

*Compared to day −7, # compared to day 0, ^ compared to CP group. All data are given as means ± SEM. *P < 0.05; ***P < 0.001 by *post hoc* ANOVA one‐way statistical analysis.

### *P. pentosaceus* LI05 administration altered the gut microbiome composition at multiple levels

The taxonomic configuration of the intestinal microbiome is altered in patients with IBD (Tamboli *et al.*, 2004). Therefore, we compared the taxonomic abundance at multiple levels among the three groups. Following DSS administration, the relative abundance of *Porphyromonadaceae, Odoribacter* and *Clostridium_sensu_stricto_1* was significantly increased in the DP group (DP vs. CP: *P* < 0.01, *P* < 0.05 and *P* < 0.001 respectively). However, the relative abundance of these taxa was decreased in the L5 group (L5 vs. DP: *P* < 0.01, *P* < 0.01 and *P* < 0.01, respectively, Fig. S3). Throughout the experiment, *P. pentosaceus* LI05 supplementation clearly altered the intestinal microbiome structure. The relative abundance of *Firmicutes* (L5 vs. DP, *P* < 0.05), *Verrucomicrobia* and *Verrucomicrobiaceae* (L5 vs. CP, *P* < 0.0001 and *P* < 0.0001) increased in the L5 group. In addition, a reduction in the relative abundance of *Bacteroidetes* and *Actinobacteria* was observed in the L5 group (*P* < 0.05 and *P* < 0.001 respectively). Furthermore, the *P. pentosaceus* LI05 treatment markedly increased the abundance of *Anaerofilum*, *Ruminiclostridium_5*, *Faecalibacterium* (L5 vs. DP, *P* < 0.01, *P* < 0.01 and *P* < 0.001 respectively; L5 vs. CP, *P* < 0.01, *P* < 0.01 and *P* < 0.01 respectively) and *Akkermansia* (L5 vs. CP, *P* < 0.0001, Figure S3), which are involved in SCFA production.

**Fig. 5:**
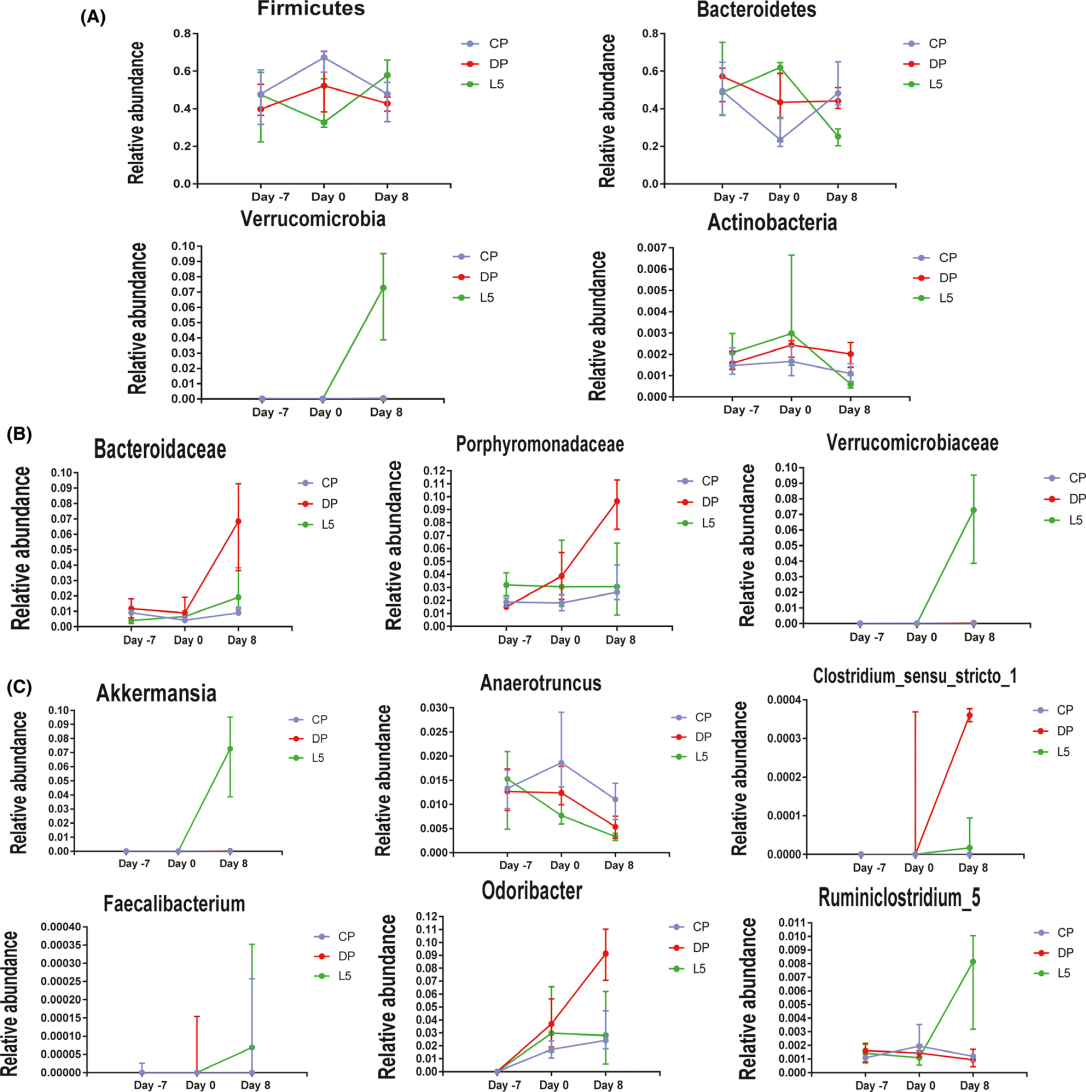
Dynamic changes in the abundance of specific taxa at the phylum (A), family (B) and genus (C) levels among the three groups during the experiment. All data are presented as medians with interquartile ranges.

Next, we analysed the dynamic alterations in the abundance of specific taxa showing marked differences among the three groups. As shown in Figure 5A, the abundance of major phyla, such as *Firmicutes,* decreased after DSS administration, whereas the *P. pentosaceus* LI05 treatment distinctly increased the abundance. The abundance of the phylum *Verrucomicrobia* did not change in the DP and CP groups over time, but showed a significant increase after the *P. pentosaceus* LI05 treatment. Additionally, the abundance of *Bacteroidaceae* and *Porphyromonadaceae* increased rapidly in the DP group. However, the curve showing the increase in the abundance of *Bacteroidaceae* in the L5 group was flat, and the abundance of *Porphyromonadaceae* even showed a decreasing trend (Fig. 5B). Importantly, the abundance of the genera *Akkermansia*, *Faecalibacterium* and *Ruminiclostridium_5* consistently increased as the time after *P. pentosaceus* LI05 supplementation increased (Fig. 5C).

We performed the LEfSe analysis of differences between groups to further investigate the prognostic microbial markers. No significant difference was observed between three groups prior to DSS administration (Figure S4). At the end of the experiment (day 8), the DP group showed a considerably altered microbiota structure, which was mainly reflected in the enrichment of the genera *Bacteroides*, *Escherichia_Shigella* and *Turicibacter* (LDA score (log_10_) > 4.8, Fig. S5A) in contrast with the CP group. In contrast, the family *Ruminococcaceae* and genus *Ruminiclostridium_9* were significantly enriched in the L5 group (LDA score (log_10_) > 4.8, Fig. S5B). Consistent with the results described above, the genus *Odoribacter* was enriched in the DP group, while the family *Verrucomicrobiaceae* and genus *Akkermansia* were enriched in the L5 group.

### The *P. pentosaceus* LI05 treatment altered the DSS‐induced changes in the faecal metabolic profiles

A non‐targeted analysis of the metabolite composition was determined in the present study to link the intestinal microbiome with metabolic functions, and 330 metabolites were identified (data not shown). The total ion stacking chromatogram of QC samples and the box plot of the metabolite intensity distribution indicated the stability and repeatability of the experiment (Fig. S6). A PCA was performed to visualize the differentially altered metabolites among the groups and showed distinctly separated clusters (Fig. S7A). An OPLS‐DA was performed, as presented in Figure S7B and C. The score plot showed a marked separation of the metabolic profiles between the groups (L5 vs. DP, Q2 cum = 0.91; DP vs. CP, Q2 cum = 0.979). The metabolites that were differentially altered by *P. pentosaceus* LI05 administration were selected using OPLS‐DA (VIP > 1, *P* < 0.05). One hundred twenty‐five selected metabolites are presented in the heat map and are mainly associated with the following pathways: amino acids, lipids, purine and carbohydrates (Figures S7D and S8). Importantly, the levels of some metabolites involved in the amino and purine pathways were significantly increased or decreased in the L5 group compared with the DP group (Fig. S7D). The levels of amino acids such as tryptophan and phenylalanine decreased after the LI05 treatment, whereas the levels of the amino acid arginine and the purine nucleoside adenosine were increased by LI05 in the present study.

### *P. pentosaceus* LI05 increased the production of SCFAs

**Fig. 6:**
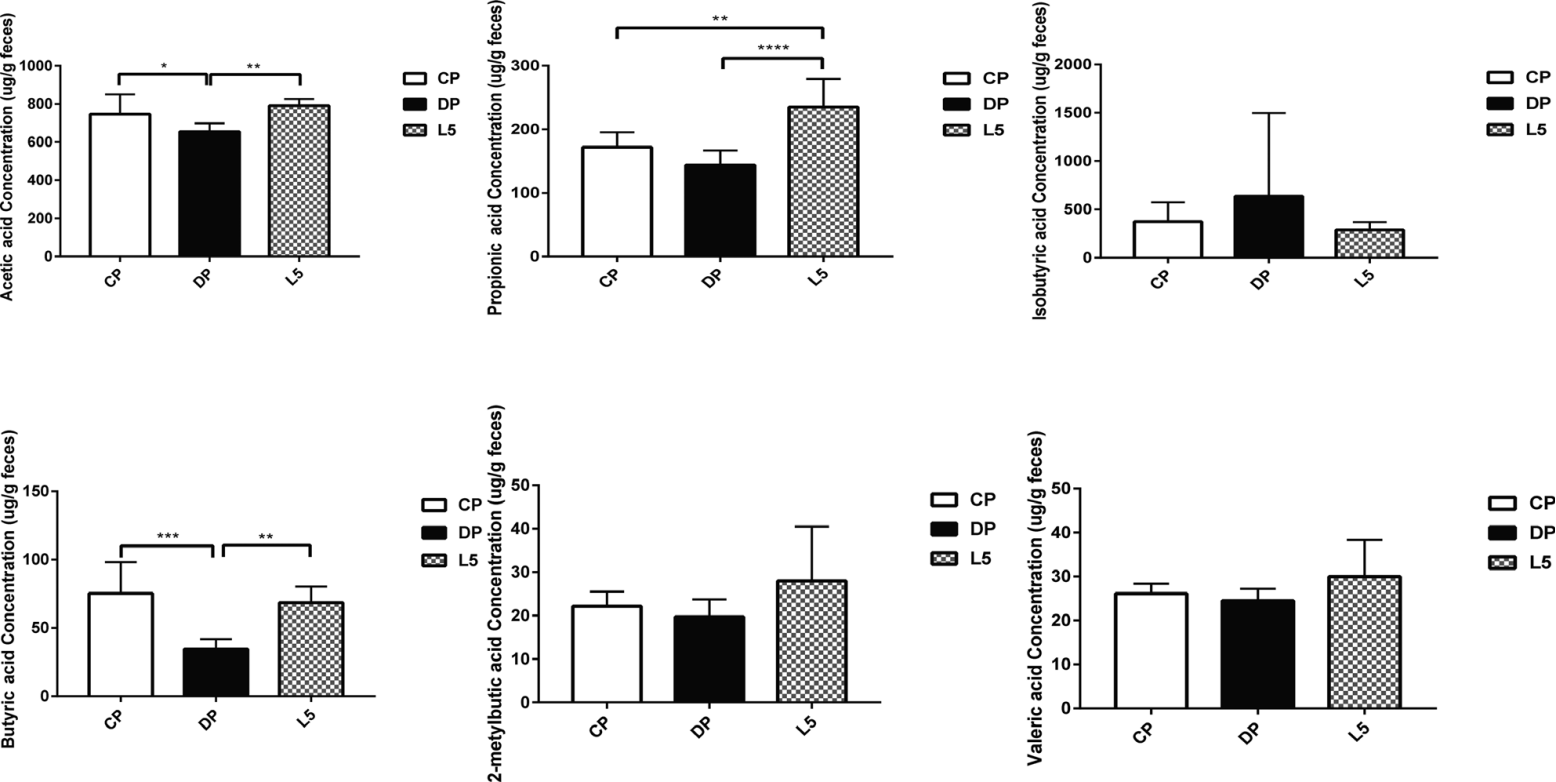
*P. pentosaceus* LI05 administration increased the production of short‐chain fatty acids (SCFAs). The faecal concentration of SCFAs was determined using GC‐MS, including acetic acid, propionic acid, isobutyric acid, butyric acid, 2‐metylbutic acid and valeric acid. All data are presented as means ± SEM. *P < 0.05, **P < 0.01, ***P < 0.001 and ****P < 0.0001 according to the post hoc one‐way ANOVA.

Significant increases in SCFA productions were observed in faecal samples from the L5 group. Six major SCFAs, acetic acid, propionic acid, isobutyric acid, butyric acid, 2‐methylbutyric acid and valeric acid, were selected and measured in the faecal samples. The acetic acid and butyric acid concentrations were remarkably decreased by DSS administration (DP vs. CP: *P* < 0.05 and *P* < 0.001, respectively, Fig. 6). In comparison, *P. pentosaceus* LI05 administration noticeably increased the levels of acetic acid, propionic acid, isobutyric acid and butyric acid (L5 vs. DP: *P* < 0.01, *P* < 0.0001 and *P* < 0.01, respectively, Fig. 6). However, no significant differences were observed in the production of isobutyric acid, 2‐methylbutyric acid and valeric acid.

### Correlations of the *P. pentosaceus* LI05‐modified gut microbiota with gut barrier markers, inflammatory cytokines and SCFAs indicate its important effect on DSS‐induced colitis

**Fig. 7:**
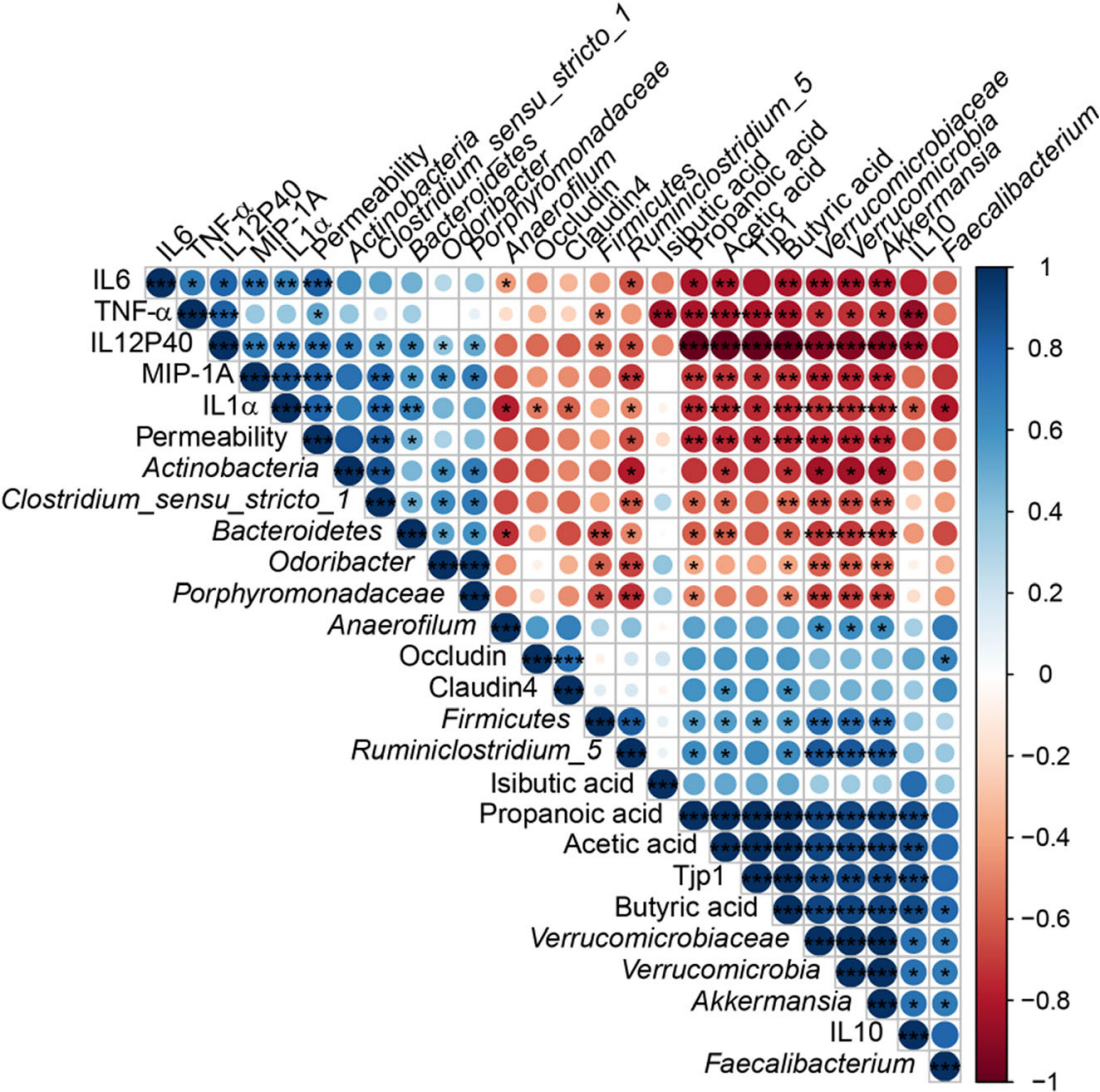
Correlations of representative microbial taxa, gut barrier markers, inflammatory cytokines and SCFAs between the DP and L5 groups. Spearman’s rank correlation coefficients were calculated. Blue indicates positive correlations, and red indicates negative correlations. Significant differences were indicated as follows: *P < 0.05, **P < 0.01 and ***P < 0.001.

Gut microbes have been shown to exert beneficial effects on IBD (Tamboli *et al.*, 2004). We conducted a correlation analysis between the altered intestinal bacteria, gut barrier markers, inflammatory cytokines and SCFAs to further explore the protective effects of the *P. pentosaceus* LI05‐modified gut microbiota. Gut bacteria, the abundances of which were markedly increased in the DP group, might be involved in the aggravation of colitis. For example, *Actinobacteria* positively correlated with TNF‐α, IL1α, IL6, IL12P40, and MIP‐1A levels and intestinal permeability (*P* < 0.05, *P* < 0.001, *P* < 0.001, *P* < 0.01, *P* < 0.001 and *P* < 0.001, respectively, Fig. 7). Similarly, *Bacteroidetes* and *Odoribacter* positively correlated with IL1α, IL12P40 and MIP‐1A levels and intestinal permeability (*P* < 0.01, *P* < 0.05, *P* < 0.01 and *P* < 0.01, respectively, Fig. 7). In contrast, the levels of these inflammatory cytokines negatively correlated with bacteria exhibiting an increased abundance after *P. pentosaceus* LI05 administration. Positive correlations were identified between IL10 levels, and almost all bacteria with markedly increased abundances in the L5 group. Specifically, *Verrucomicrobiaceae*, *Verrucomicrobia* and *Akkermansia* displayed positive correlations with the levels of gut barrier markers, such as Tjp1. In addition, the levels of SCFAs, such as acetic acid, propionic acid and butyric acid, positively correlated with *Verrucomicrobiaceae*, *Verrucomicrobia* and *Akkermansia*, whereas *Faecalibacterium* positively correlated with butyric acid levels (Fig. 7). Based on these data, the alterations in the gut microbiota induced by *P. pentosaceus* LI05 treatment play essential roles in alleviating DSS‐induced colitis by maintaining the epithelial barrier function, inhibiting inflammatory responses and reducing SCFA production.

## Discussion

Gut microbes, which are relevant to host immunity and metabolism, are key factors contributing to IBD pathogenesis and development (Tamboli *et al.*, 2004; Sartor, 2008; Marion‐Letellier *et al.*, 2016). According to multiple studies, *P. pentosaceus* LI05 exerts an important effect on protecting the host intestine by regulating the gut microbiome, thereby strengthening the gut barrier and modulating the mucosal immunity, as well as host metabolism (Bengmark *et al.*, 2011; Zhao *et al.*, 2012; Lv *et al.*, 2014b; Shi *et al.*, 2017; Xu *et al.*, 2018). Therefore, our study aimed to evaluate alterations in the gut microbiota, immune responses and metabolites (SCFA production) in a mouse model of DSS‐induced colitis after treatment with *P. pentosaceus* LI05. Mice treated with *P. pentosaceus* LI05 in the present study showed a significant amelioration of symptoms, including improvements in body weight loss, DAI score, colon length shortening and intestinal permeability. In addition, marked shifts in the compositions of the gut microbiota and metabolites were observed in mouse faecal samples, and the altered taxa might mediate dysbiosis in colitis. Intriguingly, the administration of *P. pentosaceus* LI05 increased the abundance of specific genera, such as *Akkermansia* and *Faecalibacterium*, and helped regulate the gut microbiota, reduce host inflammation and increase SCFA production.

Epithelial injury is the initiating event in DSS‐induced colitis models, and intestinal permeability is an important metric representing the severity of colitis (Wirtz *et al.*, 2017). Intestinal permeability is markedly increased after DSS exposure (Png *et al.*, 2010). Notably, probiotics increase the thickness of colonic mucus layer by regulating bacterial products (e.g. peptides and SCFAs), resulting in a strengthened intestinal barrier (Meyer‐Hoffert *et al.*, 2008; Ahl *et al.*, 2016). Our results consistently showed decreased intestinal permeability and serum LPS levels, as well as increased expression of tight junction markers after the application of *P. pentosaceus* LI05.

The recovery of intestinal permeability might be related to a reduced inflammatory response (Genser *et al.*, 2016). Based on accumulating evidence, epithelium destruction and a severe inflammatory response occur in patients with IBD (Souza *et al.*, 2005; Coskun, 2014). Similarly, the damaged intestinal epithelium induced by the administration of DSS results in increased exposure of immune cells to antigens, consequently inducing a profound immune response (Souza *et al.*, 2015; Llewellyn *et al.*, 2018). In the present study, the *P. pentosaceus* LI05 treatment exerted protective effects on colonic inflammation by decreasing the levels of the cytokines and chemokines IL1α, IL6, TNF‐α and MIP‐1A, increasing the level of the anti‐inflammatory cytokine IL10 and reducing neutrophil infiltration in the intestine. As reported in previous studies, neutrophil infiltration is observed in rectal biopsies from patients with UC, and DSS‐induced colitis is characterized by a significant increase in neutrophil accumulation in the colon (Lampinen *et al.*, 2005; Vieira *et al.*, 2013; Ranganathan *et al.*, 2013). Hence, the inhibition of neutrophil infiltration in the intestine might be an essential mechanism involved in the protection provided by probiotics in patients with IBD. The levels of the proinflammatory cytokines IL1β, IL6 and TNF‐α are increased in both animal models of colitis and patients with UC (Liu *et al.*, 2013; Lee *et al.*, 2013; Tao *et al.*, 2013). These cytokines are involved in disrupting the tight junctions of the epithelial layer through the nuclear factor (NF)‐κB signalling pathway (Liu *et al.*, 2013; Woodhouse *et al.*, 2018). In addition, interventions that block receptors of TNF‐α and IL6 markedly attenuate the progression of IBD (Hibi *et al.*, 2003). Moreover, chemokines such as MIP‐1A are generated rapidly during inflammation and have been observed to be involved in monocyte/macrophage activation (Kostova *et al.*, 2015). Remarkably, compelling evidence has shown that the level of the anti‐inflammatory cytokine IL10 is increased after probiotic treatments (e.g. *Lactobacillus* GG, VSL#3, and *Akkermansia*) in animal models of colitis (Dieleman *et al.*, 2003; Lammers *et al.*, 2003; Bian *et al.*, 2019), consistent with our findings.

The gut microbiome plays critical roles in our bodies by regulating health and disease progression (Hor *et al.*, 2019). A distinct alteration the intestinal microbiota composition occurs in patients with IBD and mice treated with DSS (Manichanh *et al.*, 2006; Frank *et al.*, 2007; Dicksved *et al.*, 2012; Putignani *et al.*, 2016), as evidenced by decreased species richness and biodiversity. A reduced diversity has also been observed in the inflamed colon within the same patient with IBD (Sepehri *et al.*, 2007). Similar results were obtained in the present study, as we observed broad intestinal microbiota dysbiosis, including reduced OTU numbers and α diversity, in mice administered DSS. Although the *P. pentosaceus* LI05 treatment did not completely reverse the altered diversity, it increased the microbial diversity in faecal samples to some extent.

The abundance of specific bacteria, such as *Enterobacteriaceae*, is increased in patients suffering from IBD and in mice with chemically induced colitis (Lupp *et al.*, 2007). *Escherichia/Shigella* and *Escherichia coli*, which belong to the family *Enterobacteriaceae*, are enriched in patients with IBD (Sokol *et al.*, 2006; Quigley *et al.*, 2013). *Escherichia/Shigella* might increase intestinal permeability and exacerbate colitis (Quigley *et al.*, 2013). Consistent with these findings, *Escherichia/Shigella* was enriched in mice treated with DSS in our study, indicating that the inflammatory environment might be conducive to the overgrowth of this bacterial clade and the exacerbation of the intestinal inflammation.

The abundance of the phylum *Firmicutes* and particularly the class Clostridia is decreased in patients IBD (Frank *et al.*, 2007). Bacteria of the class Clostridia have previously been shown to inhibit host colon inflammation by directly regulating colonic regulatory T cells (Tregs; Atarashi *et al.*, 2013). Bacteria within the class *Clostridia*, particularly the family *Ruminococcaceae* and the genus *Faecalibacterium*, play an essential role in SCFA production (Pryde *et al.*, 2002). SCFAs, which are fermented from fibre, have been observed to regulate Treg induction and inhibit neutrophil activation, which can oppose colitis (Maslowski *et al.*, 2009; Arpaia *et al.*, 2013; Furusawa *et al.*, 2013; Smith *et al.*, 2013). In particular, butyrate, one of the primary energy sources for colon cells, has been proposed to exert essential protective effects on IBD and colon cancer (Wang *et al.*, 2018). Additionally, butyrate promotes mucin production, modulates innate immune cells and suppresses NF‐κB activation, (McHardy *et al.*, 2013). *Faecalibacterium prausnitzii,* the only identified species in the genus *Faecalibacterium*, has been shown to produce butyrate to improve intestinal barrier function and maintain the immune balance in previous studies (Carlsson *et al.*, 2013). Consistent with these findings, *Faecalibacterium* was significantly enriched in mice treated with *P. pentosaceus* LI05 in our study. Moreover, butyric acid production was markedly increased after *P. pentosaceus* LI05 administration and positively correlated with an increase in *Faecalibacterium* abundance.

Notably, an increased abundance of the genus *Akkermansia* was observed in mice treated with *P. pentosaceus* LI05 in our study. Multiple studies have reported a reduced abundance of *Akkermansia* in both patients with IBD and animals with colitis (Png *et al.*, 2010; Bian *et al.*, 2019). *Akkermansia,* a mucin‐degrading bacterium, has been shown to degrade mucin into multiple metabolites, such as SCFAs, subsequently regulating host immune responses and biological functions, including glucose and lipid metabolism (Derrien *et al.*, 2004). Furthermore, *Akkermansia* promotes mucus production, maintains the intestinal barrier integrity and thereby relieves colonic mucosal inflammation (Everard *et al.*, 2013). Consistent with these findings, *Akkermansia* negatively correlated with the levels of almost all tested proinflammatory cytokines and positively correlated with the levels of intestinal barrier markers and SCFAs (acetic acid, propionic acid and butyric acid) in the present study.

Based on the evidence from compelling studies, gut microbes modulate the gastrointestinal metabolites (Rao and Samak, 2013; Hor *et al.*, 2019). Faecal amino acid levels are increased in patients with IBD (Marchesi *et al.*, 2007; Jansson *et al.*, 2009) and potentially represent biomarkers for the assessment of IBD (Hisamatsu *et al.*, 2012). Consistent with these results, the levels of the amino acids tryptophan and phenylalanine were increased after DSS exposure and decreased by *P. pentosaceus* LI05 administration. Another amino acid, arginine, can be processed into immunomodulatory metabolites (Postler and Ghosh, 2017), and its biosynthesis was increased in the L5 group. In addition, a marked difference in purine metabolism was observed between the DP and L5 groups. Adenosine is a purine nucleoside, and its levels were significantly increased after *P. pentosaceus* LI05 treatment in the present study. These findings were consistent with previous reports showing that adenosine regulates innate lymphoid cell function and alleviates colitis (Kurtz *et al.*, 2014; Crittenden *et al.*, 2018). Thus, *P. pentosaceus* LI05 administration modulates the gut microbiota by increasing its similarity to a healthy gut microbiota, and the protective effects of *P. pentosaceus* LI05 on colitis involve the ‐modification of the gut microbiota. The *P. pentosaceus* LI05‐modified gut microbiota played key roles in improving gut barrier function, reducing immune responses and promoting SCFA production.

The colitis model we used does not completely represent the clinical characteristics and pathogenesis of IBD. Clinical experiments are needed to investigate the effects of *P. pentosaceus* LI05 on humans. Although we observed a distinct distribution of the microbiota among the groups and a strong correlation between the colitis‐related and *P. pentosaceus* LI05‐modified gut microbiota, other potential mechanisms might also be involved.

Overall, the *P. pentosaceus* LI05 treatment alleviated the intestinal inflammation by maintaining gut barrier function and modulating immunological profiles, gut microbiota and metabolite compositions. As shown in the present study, *P. pentosaceus* LI05 might be applied as a potential preparation to ameliorate colitis.

## Experimental procedures

### Preparation of *P. pentosaceus* LI05

*P. pentosaceus* LI05 (CGMCC 7049), which was isolated from the faeces of healthy individuals, was cultured anaerobically overnight at 37°C in Man Rogosa Sharpe (MRS) broth (Oxoid, Thermo Fisher Biochemicals), as previously described (Shi *et al.*, 2017). Bacteria were washed with sterile phosphate‐buffered saline (PBS) and re‐suspended in PBS to a density of 1.5 × 10^10^ CFU (colony forming units)/ml before use. Two hundred microlitres of the mixture with 3 × 10^9^ CFU *P. pentosaceus* LI05 was prepared for each mouse.

### Mice and experimental colitis induction

Male C57BL/6 mice were purchased from SLAC Lab (Shanghai, China) and housed under controlled conditions (specific pathogen‐free, 12 h light and dark cycle). Animals were randomly assigned to the groups CP, DP and L5 (*n* = 8 each). Mice in the L5 (DSS + LI05) group were treated with 200 μl of *P. pentosaceus* LI05 by oral gavage from day −7 to day 6 (Fig. 1A). Mice in the CP (water + PBS) and DP (DSS + PBS) groups were treated with 200 μl of sterile PBS instead of the probiotic.

Acute colitis was induced by administering 2% (wt/vol) DSS (molecular weight: 36 000–50 000; MP Biomedicals). Briefly, mice were provided with DSS (DP and L5 groups) and drinking water (CP group) ad libitum from day 0 to day 6 (Fig. 1A). A freshly prepared DSS solution was administered every 2 days throughout our experiment. Mouse weights and faecal conditions were recorded daily; mice were humanely sacrificed on day 8; and serum and colon samples were collected. Faecal samples were collected before oral gavage (day −7), before DSS administration (day 0) and after colitis induction (day 8). Distal parts of the colon segments were measured and fixed with 10% formalin for the subsequent histological analysis. The remaining colon, serum and faecal samples were collected for subsequent procedures. The present study was approved by the ethics committee of the First Affiliated Hospital of Zhejiang University (2019‐1069).

### Disease activity index evaluation

A disease activity index (DAI) score was recorded daily after the initiation of DSS administration to further quantify the colitis severity. The DAI score (0–12) consists of three parts based on the clinical features: weight loss, faecal blood and consistency (Wirtz *et al.*, 2017). Faecal blood was assessed using a faecal occult blood reagent (Baso, BA2020E).

### Analysis of intestinal permeability and histological examination

A permeability probe, fluorescein isothiocyanate‐conjugated (FITC)–dextran (3–5 kDa, Sigma‐Aldrich), was applied for the intestinal permeability analysis, as previously described (Bian *et al.*, 2019). Animals were treated with FITC–dextran (12 mg per 20 g body weight) by gavage after an overnight fast. The FITC–dextran concentration in plasma samples was determined 4 h after oral gavage using spectrophotofluorometry (490/525 nm).

The fixed and embedded distal colon samples were then processed for haematoxylin and eosin (H&E) staining. Randomly selected slides from each group were observed by an independent pathologist using the NanoZoomer Digital Pathology system (Hamamatsu Photonics, KK, Japan). The degree of the histopathological changes was calculated by calculating the histological activity index, which consists of inflammatory cell infiltration (1–3) and the intestinal architecture (1–3), as previously reported ( Erben *et al.*, 2014).

### Immunofluorescence and immunohistochemical staining

Embedded colon sections were immunostained with antibodies (ZO1, Occludin and Ly6G), as previously described (Chung *et al.*, 2014). Images were scanned using a fluorescence confocal microscope (Zeiss, Jena, Germany). Five fields of view from each sample were selected randomly, and Image‐Pro Plus software was used to calculate the Ly6G^+^ cell ratio.

### Measurement of serum cytokine and endotoxin concentrations

The serum concentrations of cytokines and chemokines, including interleukin (IL)1α, IL6, IL10, IL12 (p40), tumour necrosis factor alpha (TNF‐α) and macrophage inflammatory protein (MIP)‐1A, were assessed using a cytokine assay kit (Bio‐Rad, CA, USA), according to the manufacturer’s instructions.

A Guduo lipopolysaccharide binding protein (LBP) ELISA kit (Shanghai, China) was used to measure the serum endotoxin levels, as previously described (Ye *et al.*, 2018).

### qRT‐PCR analysis

Total colonic RNA was extracted with an RNeasy Plus Mini kit (Qiagen, CA, USA) according to the manufacturer’s protocols. A PrimeScript RT reagent kit (Takara Biomedicals, Kusatsu, JPN) was used to reverse transcribe the RNA into cDNAs. Then, mRNA expression was determined in duplicate with SYBR Premix Ex Taq II reagent (Takara Biomedicals) using the ViiA7 real‐time PCR system (Applied Biosystems, Massachusetts, USA). All gene expression levels were assessed using the 2^−ΔΔ^ *^CT^* method and normalized to β‐actin expression. The primer sequences are provided in Table S1.

### 16S rRNA gene sequencing analysis

The 16S rRNA gene sequencing analysis was performed to determine the microbial composition, as previously reported (Bian *et al.*, 2019). Briefly, a QIAamp Fast DNA Stool Mini kit (Qiagen, Valencia, USA) was used to extract total DNA from faecal samples (200 ± 10 mg). The amplification of 16S rRNA gene was conducted using specific primers (e.g. 16S V4:515F‐806R). Following PCR and purification, an Ion Plus Fragment Library kit 48 rxns (Thermo Scientific) and an Ion S5™ XL platform were used to generate the sequencing libraries. Then, 400 bp/600 bp clean single‐end reads were obtained using a quality control (QC) procedure. Sequences displaying ≥ 97% similarity were considered as the same operational taxonomic units (OTUs).

QIIME (version 1.7.0) was used to analyse the α diversity, including the observed species, Chao1, Shannon, Simpson and ACE indices, as well as to perform the principal coordinate analysis (PCoA) of weighted and unweighted UniFrac distances. A linear discriminant analysis effect size (LefSe) analysis was conducted online (Bajaj 2014). The intergroup difference was tested using a multi‐response permutation procedure (MRPP). The correlations of bacterial taxa with colitis‐related indices and SCFA levels were determined by calculating Spearman’s rank correlation coefficients and are presented as heat maps with *P* < 0.05 generated using R software (version 2.15.3).

The 16S rRNA gene sequencing data obtained from the 72 samples in our study have been uploaded to the NCBI SRA database (PRJNA 594232).

### Analysis of the metabolic profile

Faecal metabolic profiling assessments were conducted using gas chromatography–mass spectrometry (GC‐MS). An accurately weighed faecal sample (60 mg) was mixed with 40 μl of the internal standard (2‐chloro‐l‐phenylalanine dissolved in methanol, 0.3 mg ml^−1^) and 360 μl of cold methanol. After the sample was subjected to grinding and ultrasound‐associated extraction for 30 min, 200 μl of chloroform was added and the sample was vortexed (60 Hz, 2 min). Then, 400 μl of water was added to the sample, and the sample was extracted for 30 min before an incubation at −20°C for 30 min. The sample was centrifuged (12 000 r.p.m., 10 min, 4°C), and 300 μl of the supernatant was obtained for further analysis. QC samples were obtained by pooling small aliquots of each sample and then divided into five samples. The supernatant was dried with a vacuum dryer, and 80 μl of methoxylamine hydrochloride (dissolved in pyridine, 15 mg ml^−1^) was added. The mixture was vortexed and incubated at 37°C for 90 min. Then, 80 μl of BSTFA (1% TMCS), 20 μl of n‐hexane and 10 μl of the internal standard (C8/C9/C10/C12/C14/C16, 0.8 mg ml^−1^; C18/C20/C22/C24/C26, 0.4 mg ml^−1^) were added, vortexed and incubated at 70°C for 60 min. The sample was incubated at room temperature for 30 min prior to the GC‐MS analysis. Metabolites were identified using an untargeted GC‐MS database from Lumingbio. A principal component analysis (PCA) and orthogonal partial least‐squares‐discriminant analysis (OPLS‐DA) were conducted to visualize the differences and select the differentially altered metabolites among the groups; metabolites with a variable importance in the projection (VIP) value > 1 were selected as significantly different.

SCFAs in faeces (20 mg) were extracted and mixed with 500 μl of water containing 10 μg ml^−1^ hexanoic acid‐d3 as an internal control. Then, the samples were vortexed, centrifuged (15 000× r.p.m., 5 min) and mixed with 500 μl of ethyl acetate (5% concentrated sulfuric acid). Following a second centrifugation, the supernatant was incubated at 4°C for 30 min and transferred to specimen tubes for further analysis. Standard mixtures of six SCFAs were prepared using the same procedures used for the samples.

### Statistical analysis

The data are presented as the means ± SEM or medians with interquartile ranges. The Kolmogorov–Smirnov test was used to analyse the normality of the data. For most data, the Kruskal–Wallis test (for data with non‐normal distributions) or one‐way ANOVA with Bonferroni’s post hoc test (for data with normal distributions) was used to determine the significance between three groups. Analyses of colon length, LBP, FITC–dextran, Tjp1, Occludin, Claudin 4, CB1, CB2, serum cytokine levels, α‐diversity and metabolite levels were performed using one‐way ANOVA with Bonferroni’s post hoc test. Body weight loss was analysed using an unpaired t test followed by Welch’s corrections. DAI, histopathological score and taxonomic relative abundance were assessed using the Kruskal–Wallis test. A LefSe analysis was conducted between three groups and at three time points (day −7, day 0 and day 8). Statistical analyses were conducted using R software (version 2.15.3) and SPSS (version 20.0). Images were constructed using GraphPad Prism (version 7.0) and R software (version 2.15.3). Two‐tailed *P* values < 0.05 were considered statistically significant.

## Conflicts of interest

The authors declare no conflicts of interest.

## Authors’ contributions

Bian, Yang, Wu, Li, Jiang and Fang designed and performed the experiments; Bian, Lv, Wang, Lu, Xie, Xia and Shi analysed the data; and Bian, Ye and Li wrote and reviewed the manuscript.

## Supporting information


**Fig. S1**. Species accumulation box plot depicting the species richness in the three groups on days −7, 0 and 8.
**Fig. S2**. PCoA plot based on the weighted UniFrac distances among three groups on day −7 (left panel) and day 0 (right panel). Each point represents a sample. Adonis was used to test for microbial community clustering using weighted UniFrac distance matrices.
**Fig. S3**. Relative abundance of taxa at the phylum (A), family (B) and genus (C) levels. Bar charts present the differences in the abundance of specific taxa among the three groups (day 8). All data are presented as medians with interquartile ranges. *P < 0.05, **P < 0.01, ***P < 0.001 and ****P < 0.0001 according to the Kruskal–Wallis tests.
**Fig. S4**. LEfSe analysis comparing differences between three groups on day −7 (A) and day 0 (B).
**Fig. S5**. The *P. pentosaceus* LI05 treatment altered the gut microbial composition. (A) The LEfSe cladogram represents taxa enriched in the CP (red) and DP (green) groups (left panel) and discriminative biomarkers with an LDA score > 4.8 (left panel) between two groups (day 8). (B) The LEfSe cladogram represents taxa enriched in the DP (red) and L5 (green) groups (left panel) and discriminative biomarkers with an LDA score > 4.8 (left panel) between two groups.
**Fig. S6**. (A) The total ion stacking chromatogram of QC samples showed the small variation caused by instrument error. (B) The box plot of the metabolite intensity distribution.
**Fig. S7** **. **The *P. pentosaceus* LI05 treatment altered DSS‐induced faecal metabolic profiles. (A) PCA plot comparing the QC (green), CP (blue), DP (red), L5 (yellow) groups. (B) OPLS‐DA score plot comparing the CP (blue) and DP (red) groups. (C) OPLS‐DA score plot comparing the L5 (yellow) and DP (red) groups. (D) Heat map showing the distribution of different levels of differentially altered metabolites between the L5 and DP groups based on the hierarchical clustering analysis. In the heat map profiles, relative values normalized to 3 and − 3 are represented by different colours. Red indicates the high levels of differentially altered metabolites, and green indicates low levels of differentially altered metabolites.
**Fig. S8**. (A) Map of the top 10 metabolic pathways enriched in the L5 and DP groups. The red line indicates a *P* value = 0.01, and the blue line indicates a *P* value = 0.05. (B) Bubble diagram of the top 20 metabolic pathways enriched in the L5 and DP groups. Enrichment factor = number of the significantly different metabolites/total metabolites in the pathway. The colour change from red to green indicates a decrease in the *P* value. A larger bubble indicates a greater number of metabolites that were enriched in the pathway.
**Table S1**. Specific primers used for the RT‐PCR analyses.
**Table S2**. MRPP test used to analyze the β diversity in fecal analysis.

## References

[mbt213583-bib-0001] Ahl, D. , Liu, H. , Schreiber, O. , Roos, S. , Phillipson, M. , and Holm, L. (2016) *Lactobacillus reuteri* increases mucus thickness and ameliorates dextran sulphate sodium‐induced colitis in mice. Acta Physiol (Oxf) 217: 300–310. 27096537 10.1111/apha.12695

[mbt213583-bib-0002] Arpaia, N. , Campbell, C. , Fan, X. , Dikiy, S. , van der Veeken, J. , deRoos, P. *, et al* (2013) Metabolites produced by commensal bacteria promote peripheral regulatory T‐cell generation. Nature 504: 451–455. 24226773 10.1038/nature12726 PMC3869884

[mbt213583-bib-0003] Atarashi, K. , Tanoue, T. , Oshima, K. , Suda, W. , Nagano, Y. , Nishikawa, H. , *et al* (2013) Treg induction by a rationally selected mixture of Clostridia strains from the human microbiota. Nature 500: 232–236. 23842501 10.1038/nature12331

[mbt213583-bib-0004] Bengmark, S. , Di Cocco, P. , Clemente, K. , Corona, L. , Angelico, R. , and Manzia, T. , *et al* (2011) Bio‐ecological control of chronic liver disease and encephalopathy. Minerva Med 102: 309–319. 21959704

[mbt213583-bib-0005] Bian, X. , Wu, W. , Yang, L. , Lv, L. , Wang, Q. , Li, Y. , *et al* (2019) Administration of *Akkermansia muciniphila* Ameliorates Dextran sulfate sodium‐induced ulcerative colitis in mice. Front Microbiol 10: 2259. 31632373 10.3389/fmicb.2019.02259 PMC6779789

[mbt213583-bib-0006] Bibiloni, R. , Fedorak, R.N. , Tannock, G.W. , Madsen, K.L. , Gionchetti, P. , Campieri, M. , *et al* (2005) VSL#3 probiotic‐mixture induces remission in patients with active ulcerative colitis. Am J Gastroenterol 100: 1539–1546. 15984978 10.1111/j.1572-0241.2005.41794.x

[mbt213583-bib-0007] Blander, J.M. , Longman, R.S. , Iliev, I.D. , Sonnenberg, G.F. , and Artis, D. (2017) Regulation of inflammation by microbiota interactions with the host. Nat Immunol 18: 851–860. 28722709 10.1038/ni.3780 PMC5800875

[mbt213583-bib-0008] Carlsson, A.H. , Yakymenko, O. , Olivier, I. , Natividad, J.M. , Jury, J. , and Lu, J. , *et al* (2013) *Faecalibacterium prausnitzii* supernatant improves intestinal barrier function in mice DSS colitis. Scand J Gastroenterol 48: 1136–1144. 23971882 10.3109/00365521.2013.828773

[mbt213583-bib-0009] Chen, R.C. , Xu, L.M. , Du, S.J. , Huang, S.S. , Wu, H. , Dong, J.J. , *et al* (2016) *Lactobacillus rhamnosus* GG supernatant promotes intestinal barrier function, balances Treg and TH17 cells and ameliorates hepatic injury in a mouse model of chronic‐binge alcohol feeding. Toxicol Lett 241: 103–110. 26617183 10.1016/j.toxlet.2015.11.019

[mbt213583-bib-0010] Chung, C.Y. , Alden, S.L. , and Funderburg, N.T. , Fu, P. , and Levine, A.D. (2014) Progressive proximal‐to‐distal reduction in expression of the tight junction complex in colonic epithelium of virally‐suppressed HIV plus individuals. PLoS Pathog 10: e1004198. 24968145 10.1371/journal.ppat.1004198 PMC4072797

[mbt213583-bib-0011] Coskun, M. (2014) Intestinal epithelium in inflammatory bowel disease. Front Med 1: 24. 10.3389/fmed.2014.00024 PMC4292184 25593900

[mbt213583-bib-0012] Crittenden, S. , Cheyne, A. , Adams, A. , Forster, T. , Robb, C.T. , Felton, J. (2018) Purine metabolism controls innate lymphoid cell function and protects against intestinal injury. Immunol Cell Biol 96: 1049–1059. 29758102 10.1111/imcb.12167 PMC6248310

[mbt213583-bib-0013] Derrien, M. , Vaughan, E.E. , Plugge, C.M. , de Vos, W.M. (2004) *Akkermansia muciniphila* gen. nov., sp nov., a human intestinal mucin‐degrading bacterium. Int J Syst Evol Microbiol 54: 1469–1476. 15388697 10.1099/ijs.0.02873-0

[mbt213583-bib-0014] Dicksved, J. , Schreiber, O. , Willing, B. , Petersson, J. , Rang, S. , Phillipson, M. , *et al* (2012) *Lactobacillus reuteri* maintains a functional mucosal barrier during DSS treatment despite mucus layer dysfunction. PLoS ONE 7: e46399. 23029509 10.1371/journal.pone.0046399 PMC3459901

[mbt213583-bib-0015] Dieleman, L.A. , Goerres, M.S. , Arends, A. , Sprengers, D. , Torrice, C. , Hoentjen, F. , *et al* (2003) Lactobacillus GG prevents recurrence of colitis in HLA‐B27 transgenic rats after antibiotic treatment. Gut 52: 370–376. 12584218 10.1136/gut.52.3.370 PMC1773552

[mbt213583-bib-0016] Erben, U. , Loddenkemper, C. , Doerfel, K ., Spieckermann, S. , Haller, D. , Heimesaat, M.M. , *et al* (2014) A guide to histomorphological evaluation of intestinal inflammation in mouse models. Int J Clin Exp Pathol 7: 4557–4576. 25197329 PMC4152019

[mbt213583-bib-0017] Everard, A. , Belzer, C. , Geurts, L. , Ouwerkerk, J.P. , Druart, C. , Bindels, L.B. , *et al* (2013) Cross‐talk between Akkermansia muciniphila and intestinal epithelium controls diet‐induced obesity. Proc Natl Acad Sci USA 110: 9066–9071. 23671105 10.1073/pnas.1219451110 PMC3670398

[mbt213583-bib-0018] Frank, D.N. , Amand, A.L.S. , Feldman, R.A. , Boedeker, E.C. , Harpaz, N. , and Pace, N.R. (2007) Molecular‐phylogenetic characterization of microbial community imbalances in human inflammatory bowel diseases. Proc Natl Acad Sci USA 104: 13780–13785. 17699621 10.1073/pnas.0706625104 PMC1959459

[mbt213583-bib-0019] Franzosa, E.A. , Sirota‐Madi, A. , Avila‐Pacheco, J. , Fornelos, N. , Haiser, H.J. , Reinker, S. , *et al* (2019) Gut microbiome structure and metabolic activity in inflammatory bowel disease. Nature Microbiol 4: 293–305. 30531976 10.1038/s41564-018-0306-4 PMC6342642

[mbt213583-bib-0020] Furusawa, Y. , Obata, Y. , Fukuda, S. , Endo, T.A. , Nakato, G. , Takahashi, D. , *et al* (2013) Commensal microbe‐derived butyrate induces the differentiation of colonic regulatory T cells. Nature 504: 446–450. 24226770 10.1038/nature12721

[mbt213583-bib-0021] Genser, L. , Poitou, C. , Brot‐Laroche, E ., Rousset, M. , Vaillant, J.C. , Clément, K. , *et al* (2016) Alteration of intestinal permeability: the missing link between gut microbiota modifications and inflammation in obesity? Med Sci 32: 461–469. 10.1051/medsci/20163205012 27225918

[mbt213583-bib-0022] Hibi, T. , Inoue, N. , Ogata, H. , and Naganuma, M. (2003) Introduction and overview: recent advances in the immunotherapy of inflammatory bowel disease. J Gastroenterol 38(Suppl 15): 36–42. 12698869

[mbt213583-bib-0023] Hisamatsu, T. , Okamoto, S. , Hashimoto, M. , Muramatsu, T. , Andou, A. , Uo, M. , *et al* (2012) Novel, objective, multivariate biomarkers composed of plasma amino acid profiles for the diagnosis and assessment of inflammatory bowel disease. PLoS ONE 7: e31131. 22303484 10.1371/journal.pone.0031131 PMC3269436

[mbt213583-bib-0024] Hor, Y.Y. , Lew, L.C. , and Jaafar, M.H. , Lau, A.S.Y. , Ong, J.S. , Kato, T. , *et al* (2019) *Lactobacillus* sp. improved microbiota and metabolite profiles of aging rats. Pharmacol Res 146: 104312. 31207344 10.1016/j.phrs.2019.104312

[mbt213583-bib-0025] Jansson, J. , Willing, B. , Lucio, M. , Fekete, A. , Dicksved, J. , Halfvarson, J. , *et al* (2009) Metabolomics reveals metabolic biomarkers of Crohn's disease. PLoS ONE 4: e6386. 19636438 10.1371/journal.pone.0006386 PMC2713417

[mbt213583-bib-0026] Kostova, Z. , Batsalova, T. , Moten, D. , Teneva, I. , and Dzhambazov, B. (2015) Ragweed‐allergic subjects have decreased serum levels of chemokines CCL2, CCL3, CCL4 and CCL5 out of the pollen season. Cent Eur J Immunol 40: 442–446. 26862308 10.5114/ceji.2015.56965 PMC4737740

[mbt213583-bib-0027] Kurtz, C.C. , Drygiannakis, I. , Naganuma, M. , Feldman, S. , Bekiaris, V. , Linden, J. , *et al* (2014) Extracellular adenosine regulates colitis through effects on lymphoid and nonlymphoid cells. Am J Physiol Gastrointest Liver Physiol 307: G338–G346. 24875104 10.1152/ajpgi.00404.2013 PMC4121634

[mbt213583-bib-0028] Lammers, K.M. , Brigidi, P. , Vitali, B. , Gionchetti, P. , Rizzello, F. , Caramelli, E. , *et al* (2003) Immunomodulatory effects of probiotic bacteria DNA: IL‐1 and IL‐10 response in human peripheral blood mononuclear cells. FEMS Immunol Med Microbiol 38: 165–172. 13129651 10.1016/S0928-8244(03)00144-5

[mbt213583-bib-0029] Lampinen, M. , Ronnblom, A. , Amin, K. , Kristjansson, G. , Rorsman, F. , Sangfelt, P. ,*et al* (2005) Eosinophil granulocytes are activated during the remission phase of ulcerative colitis. Gut 54: 1714–1720. 15886302 10.1136/gut.2005.066423 PMC1774808

[mbt213583-bib-0030] Lane, E.R. , Zisman, T.L. , and Suskind, D.L. (2017) The microbiota in inflammatory bowel disease: current and therapeutic insights. J Inflamm Res 10: 63–73. 28652796 10.2147/JIR.S116088 PMC5473501

[mbt213583-bib-0031] Lee, H.J. , Lee, H.G. , Choi, K.S. , Surh, Y.J. , and Na, H.K. (2013) Diallyl trisulfide suppresses dextran sodium sulfate‐induced mouse colitis: NF‐kappaB and STAT3 as potential targets. Biochem Biophys Res Commun 437: 267–273. 23811270 10.1016/j.bbrc.2013.06.064

[mbt213583-bib-0032] Liu, L. , Liu, Y.L. , Liu, G.X. , Chen, X. , Yang, K. , Yang, Y.X. , *et al* (2013) Curcumin ameliorates dextran sulfate sodium‐induced experimental colitis by blocking STAT3 signaling pathway. Int Immunopharmacol 17: 314–320. 23856612 10.1016/j.intimp.2013.06.020

[mbt213583-bib-0033] Liu, M. , Sun, T. , Li, N. , Peng, J. , Fu, D. , Li, W. , *et al* (2019) BRG1 attenuates colonic inflammation and tumorigenesis through autophagy‐dependent oxidative stress sequestration. Nat Commun 10: 4614. 31601814 10.1038/s41467-019-12573-z PMC6787222

[mbt213583-bib-0034] Llewellyn, S.R. , Britton, G.J. , Contijoch, E.J. , Vennaro, O.H. , Mortha, A. , Colombel, J.F. , *et al* (2018) Interactions between diet and the intestinal microbiota alter intestinal permeability and colitis severity in mice. Gastroenterology 154: 1037–1046.e2. 29174952 10.1053/j.gastro.2017.11.030 PMC5847454

[mbt213583-bib-0035] Lupp, C. , Robertson, M.L. , Wickham, M.E ., Sekirov, I. , Champion, O.L. , Gaynor, E.C. , *et al* (2007) Host‐mediated inflammation disrupts the intestinal microbiota and promotes the overgrowth of Enterobacteriaceae (Vol 2, pg 119, 2007). Cell Host Microbe 2: 204. 18030708 10.1016/j.chom.2007.08.002

[mbt213583-bib-0036] Lv, L.X. , Li, Y.D. , Hu, X.J. , Shi, H.Y. , and Li, L.J. (2014a) Whole‐genome sequence assembly of *Pediococcus pentosaceus* LI05 (CGMCC 7049) from the human gastrointestinal tract and comparative analysis with representative sequences from three food‐borne strains. Gut Pathog 6: 36. 25349631 10.1186/s13099-014-0036-y PMC4209512

[mbt213583-bib-0037] Lv, L.X. , Hu, X.J. , Qian, G.R. , Zhang, H. , Lu, H.F. , Zheng, B.W. , *et al* (2014b) Administration of *Lactobacillus salivarius* LI01 or *Pediococcus pentosaceus* LI05 improves acute liver injury induced by D‐galactosamine in rats. Appl Microbiol Biotechnol 98: 5619–5632. 24639205 10.1007/s00253-014-5638-2

[mbt213583-bib-0038] Manichanh, C. , Rigottier‐Gois, L. , Bonnaud, E. , Gloux, K. , Pelletier, E. , Frangeul, L. , *et al* (2006) Reduced diversity of faecal microbiota in Crohn's disease revealed by a metagenomic approach. Gut 55: 205–211. 16188921 10.1136/gut.2005.073817 PMC1856500

[mbt213583-bib-0039] Mantegazza, C. , Molinari, P. , D'Auria, E. , Sonnino, M. , Morelli, L. , and Zuccotti, G.V. (2018) Probiotics and antibiotic‐associated diarrhea in children: a review and new evidence on *Lactobacillus rhamnosus* GG during and after antibiotic treatment. Pharmacol Res 128: 63–72. 28827186 10.1016/j.phrs.2017.08.001

[mbt213583-bib-0040] Marchesi, J.R. , Holmes, E. , Khan, F. , Kochhar, S. , Scanlan, P. , Shanahan, F. , *et al* (2007) Rapid and noninvasive metabonomic characterization of inflammatory bowel disease. J Proteome Res 6: 546–551. 17269711 10.1021/pr060470d

[mbt213583-bib-0041] Marion‐Letellier, R. , Savoye, G. , and Ghosh, S. (2016) IBD. In food we trust. J Crohns Colitis 10: 1351–1361. 27194533 10.1093/ecco-jcc/jjw106

[mbt213583-bib-0042] Maslowski, K.M. , Vieira, A.T. , Ng, A. , Kranich, J. , Sierro, F. , Yu, D. , *et al* (2009) Regulation of inflammatory responses by gut microbiota and chemoattractant receptor GPR43. Nature 461: 1282–1286. 19865172 10.1038/nature08530 PMC3256734

[mbt213583-bib-0043] McHardy, I.H. , Goudarzi, M. , Tong, M. , Ruegger, P.M. , Schwager, E. , Weger, J.R. , *et al* (2013) Integrative analysis of the microbiome and metabolome of the human intestinal mucosal surface reveals exquisite inter‐relationships. Microbiome 1: 17. 24450808 10.1186/2049-2618-1-17 PMC3971612

[mbt213583-bib-0044] Meyer‐Hoffert, U. , Hornef, M.W. , Henriques‐Normark, B. , *et al* (2008) Secreted enteric antimicrobial activity localises to the mucus surface layer. Gut 57: 764–771. 18250125 10.1136/gut.2007.141481

[mbt213583-bib-0045] Png, C.W. , Linden, S.K. , Gilshenan, K.S. , Zoetendal, E.G. , McSweeney, C.S. , Sly, L.I. , *et al* (2010) Mucolytic bacteria with increased prevalence in IBD mucosa augment in vitro utilization of mucin by other bacteria. Am J Gastroenterol 105: 2420–2428. 20648002 10.1038/ajg.2010.281

[mbt213583-bib-0046] Postler, T.S. , and Ghosh, S. (2017) Understanding the Holobiont: how microbial metabolites affect human health and shape the immune system. Cell Metab 26: 110–130. 28625867 10.1016/j.cmet.2017.05.008 PMC5535818

[mbt213583-bib-0047] Pryde, S.E. , Duncan, S.H. , Hold, G.L. , Stewart, C.S. , and Flint, H.J. (2002) The microbiology of butyrate formation in the human colon. FEMS Microbiol Lett 217: 133–139. 12480096 10.1111/j.1574-6968.2002.tb11467.x

[mbt213583-bib-0048] Putignani, L. , Del Chierico, F. , Vernocchi, P. , Cicala, M. , Cucchiara, S. , and Dallapiccola, B. (2016) Gut microbiota dysbiosis as risk and premorbid factors of IBD and IBS along the childhood‐adulthood transition. Inflamm Bowel Dis 22: 487–504. 26588090 10.1097/MIB.0000000000000602

[mbt213583-bib-0049] Qiu, X. , Zhang, M. , Yang, X. , Hong, N. , and Yu, C. (2013) Faecalibacterium prausnitzii upregulates regulatory T cells and anti‐inflammatory cytokines in treating TNBS‐induced colitis. J Crohns Colitis 7: e558–e568. 23643066 10.1016/j.crohns.2013.04.002

[mbt213583-bib-0050] Quigley, E.M.M. , Stanton, C. , and Murphy, E.F. (2013) The gut microbiota and the liver. Pathophysiological and clinical implications. J Hepatol 58: 1020–1027. 23183530 10.1016/j.jhep.2012.11.023

[mbt213583-bib-0051] Ranganathan, P. , Jayakumar, C. , Manicassamy, S. , and Ramesh, G. (2013) CXCR2 knockout mice are protected against DSS‐colitis‐induced acute kidney injury and inflammation. Am J Physiol Renal Physiol 305: F1422–F1427. 23986515 10.1152/ajprenal.00319.2013 PMC3840251

[mbt213583-bib-0052] Rao, R.K. , and Samak, G. (2013) Protection and restitution of gut barrier by probiotics: nutritional and clinical implications. Curr Nutr Food Sci 9: 99–107. 24353483 10.2174/1573401311309020004 PMC3864899

[mbt213583-bib-0053] Rossi, O. , Khan, M.T. , Schwarzer, M. , Hudcovic, T. , Srutkova, D. , Duncan, S.H. , *et al* (2015) Faecalibacterium prausnitzii Strain HTF‐F and its extracellular polymeric matrix attenuate clinical parameters in DSS‐induced colitis. PLoS ONE 10: e0123013. 25910186 10.1371/journal.pone.0123013 PMC4409148

[mbt213583-bib-0054] Sartor, R.B. (2008) Microbial influences in inflammatory bowel diseases. Gastroenterology 134: 577–594. 18242222 10.1053/j.gastro.2007.11.059

[mbt213583-bib-0055] Sepehri, S. , Kotlowski, R. , Bernstein, C.N. , and Krause, D.O. (2007) Microbial diversity of inflamed and noninflamed gut biopsy tissues in inflammatory bowel disease. Inflamm Bowel Dis 13: 675–683. 17262808 10.1002/ibd.20101

[mbt213583-bib-0056] Shi, D. , Lv, L. , Fang, D. , Wu, W. , Hu, C. , Xu, L. , *et al* (2017) Administration of *Lactobacillus salivarius* LI01 or Pediococcus pentosaceus LI05 prevents CCl(4)‐induced liver cirrhosis by protecting the intestinal barrier in rats. Sci Rep 7: 6927. 28761060 10.1038/s41598-017-07091-1 PMC5537250

[mbt213583-bib-0057] Smith, P.M. , Howitt, M.R. , Panikov, N. , Michaud, M. , Gallini, C.A. , Bohlooly, M. , *et al* (2013) The microbial metabolites, short‐chain fatty acids, regulate colonic Treg cell homeostasis. Science 341: 569–573. 23828891 10.1126/science.1241165 PMC3807819

[mbt213583-bib-0058] Sokol, H. , Lepage, P. , Seksik, P. , Dore, J. , and Marteau, P. (2006) Temperature gradient gel electrophoresis of fecal 16S rRNA reveals active *Escherichia coli* in the microbiota of patients with ulcerative colitis. J Clin Microbiol 44: 3172–3177. 16954244 10.1128/JCM.02600-05 PMC1594675

[mbt213583-bib-0059] Sokol, H. , Seksik, P. , Furet, J.P. , Firmesse, O. , Nion‐Larmurier, I. , Beaugerie, L. , *et al* (2009) Low counts of Faecalibacterium prausnitzii in colitis microbiota. Inflamm Bowel Dis 15: 1183–1189. 19235886 10.1002/ibd.20903

[mbt213583-bib-0060] Souza, H.S. , Tortori, C.J. , Castelo‐Branco, M.T. , Carvalho, A.T.P. , Margallo, V.S. , Delgado, C.F.S. , *et al* (2005) Apoptosis in the intestinal mucosa of patients with inflammatory bowel disease: evidence of altered expression of FasL and perforin cytotoxic pathways. Int J Colorectal Dis 20: 277–286. 15503066 10.1007/s00384-004-0639-8

[mbt213583-bib-0061] Souza, D.G. , Senchenkova, E.Y. , Russell, J. , and Granger, D.N. (2015) MyD88 mediates the protective effects of probiotics against the arteriolar thrombosis and leukocyte recruitment associated with experimental colitis. Inflamm Bowel Dis 21: 888–900. 25738377 10.1097/MIB.0000000000000331 PMC4366293

[mbt213583-bib-0062] Tamboli, C.P. , Neut, C. , Desreumaux, P. , and Colombel, J.F. (2004) Dysbiosis as a prerequisite for IBD. Gut 53: 1057. PMC1774115 15194668

[mbt213583-bib-0063] Tao, F. , Qian, C. , Guo, W. , Luo, Q. Xu, Q. , and Sun, Y. (2013) Inhibition of Th1/Th17 responses via suppression of STAT1 and STAT3 activation contributes to the amelioration of murine experimental colitis by a natural flavonoid glucoside icariin. Biochem Pharmacol 85: 798–807. 23261528 10.1016/j.bcp.2012.12.002

[mbt213583-bib-0064] Vemuri, R.C. , Gundamaraju, R. , Shinde, T. , and Eri, R. (2017) Therapeutic interventions for gut dysbiosis and related disorders in the elderly: antibiotics, probiotics or faecal microbiota transplantation? Benef Microbes 8: 179–192. 28008784 10.3920/BM2016.0115

[mbt213583-bib-0065] Vemuri, R. , Gundamaraju, R. , Shastri, M.D. , Shukla, S.D. , Kalpurath, K. , Ball, M. , *et al* (2018) Gut microbial changes, interactions, and their implications on human lifecycle: an ageing perspective. Biomed Res Int 2018: 4178607. 29682542 10.1155/2018/4178607 PMC5846367

[mbt213583-bib-0066] Vieira, A.T. , Teixeira, M.M. , and Martins, F.S. (2013) The role of probiotics and prebiotics in inducing gut immunity. Front Immunol 4: 445. 24376446 10.3389/fimmu.2013.00445 PMC3859913

[mbt213583-bib-0067] Wang, Y. , Guo, Y. , Chen, H ., Wei, H. , and Wan, C. (2018) Potential of *Lactobacillus plantarum* ZDY2013 and Bifidobacterium bifidum WBIN03 in relieving colitis by gut microbiota, immune, and anti‐oxidative stress. Can J Microbiol 64: 327–337. 29401402 10.1139/cjm-2017-0716

[mbt213583-bib-0068] Wirtz, S. , Popp, V. , Kindermann, M. , Gerlach, K. , Weigmann, B. , Fichtner‐Feigl, S. , *et al* (2017) Chemically induced mouse models of acute and chronic intestinal inflammation. Nat Protoc 12: 1295–1309. 28569761 10.1038/nprot.2017.044

[mbt213583-bib-0069] Wlodarska, M. , Kostic, A.D. , and Xavier, R.J. (2015) An integrative view of microbiome‐host interactions in inflammatory bowel diseases. Cell Host Microbe 17: 577–591. 25974300 10.1016/j.chom.2015.04.008 PMC4498258

[mbt213583-bib-0070] Woodhouse, C.A. , Patel, V.C. , Singanayagam, A. , and Shawcross, D.l. (2018) Review article: the gut microbiome as a therapeutic target in the pathogenesis and treatment of chronic liver disease. Aliment Pharmacol Ther 47: 192–202. 29083037 10.1111/apt.14397

[mbt213583-bib-0071] Wu, W. , Lv, L. , Shi, D. , Ye, J. , Fang, D. , Guo, F. , *et al* (2017) Protective effect of *Akkermansia muciniphila* against immune‐mediated liver injury in a mouse model. Front Microbiol 8: 1804. 29033903 10.3389/fmicb.2017.01804 PMC5626943

[mbt213583-bib-0072] Xu, Q. , Gu, S. , Chen, Y. , Quan, J. , Lv, L. , Chen, D. , *et al* (2018) Protective effect of Pediococcus pentosaceus LI05 against *Clostridium difficile* infection in a mouse model. Front Microbiol 9: 2396. 30356740 10.3389/fmicb.2018.02396 PMC6189400

[mbt213583-bib-0073] Ye, J. , Lv, L. , Wu, W. , Li, Y. , Shi, D. , Fang, D. , *et al* (2018) Butyrate protects mice against methionine‐choline‐deficient diet‐induced non‐alcoholic steatohepatitis by improving gut barrier function, attenuating inflammation and reducing endotoxin levels. Front Microbiol 9: 1967. 30186272 10.3389/fmicb.2018.01967 PMC6111843

[mbt213583-bib-0074] Zhao, X. , Higashikawa, F. , Noda, M. , Kawamura, Y. , Matoba, Y. , Kumagai, T. , and Sugiyama, M. (2012) The obesity and fatty liver are reduced by plant‐derived *Pediococcus pentosaceus* LP28 in high fat diet‐induced obese mice. PLoS ONE 7: e30696. 22363472 10.1371/journal.pone.0030696 PMC3281851

