# Supplementary material for: Pediococcus pentosaceus LI05 alleviates DSS‐induced colitis by modulating immunological profiles, the gut microbiota and short‐chain fatty acid levels in a mouse model
Source: Microb Biotechnol. 2020 May 3;13(4):1228–44. doi: 10.1111/1751-7915.13583 (PMC7264873; doi:10.1111/1751-7915.13583)
Supplement: Supplementary file 1 — Fig. S1. Species accumulation box plot depicting the species richness in the three groups on days −7, 0 and 8. Fig. S2. PCoA plot based on the weighted UniFrac distances among three groups on day −7 (left panel) and day 0 (right panel). Each point represents a sample. Adonis was used to test for microbial community clustering using weighted UniFrac distance matrices. Fig. S3. Relative abundance of taxa at the phylum (A), family (B) and genus (C) levels. Bar charts present the differences in the abundance of specific taxa among the three groups (day 8). All data are presented as medians with interquartile ranges. *P < 0.05, **P < 0.01, ***P < 0.001 and ****P < 0.0001 according to the Kruskal–Wallis tests. Fig. S4. LEfSe analysis comparing differences between three groups on day −7 (A) and day 0 (B). Fig. S5. The P. pentosaceus LI05 treatment altered the gut microbial composition. (A) The LEfSe cladogram represents taxa enriched in the CP (red) and DP (green) groups (left panel) and discriminative biomarkers with an LDA score > 4.8 (left panel) between two groups (day 8). (B) The LEfSe cladogram represents taxa enriched in the DP (red) and L5 (green) groups (left panel) and discriminative biomarkers with an LDA score > 4.8 (left panel) between two groups. Fig. S6. (A) The total ion stacking chromatogram of QC samples showed the small variation caused by instrument error. (B) The box plot of the metabolite intensity distribution. Fig. S7 . The P. pentosaceus LI05 treatment altered DSS‐induced faecal metabolic profiles. (A) PCA plot comparing the QC (green), CP (blue), DP (red), L5 (yellow) groups. (B) OPLS‐DA score plot comparing the CP (blue) and DP (red) groups. (C) OPLS‐DA score plot comparing the L5 (yellow) and DP (red) groups. (D) Heat map showing the distribution of different levels of differentially altered metabolites between the L5 and DP groups based on the hierarchical clustering analysis. In the heat map profiles, relative values normalized to 3 and − 3 ar [file MBT2-13-1228-s001.docx]

**Supplementary Table S1. Specific primers used for the RT-PCR** **analyses.**

| **Gene** | **Forward Sequence (5 ' - 3 ' )** | **Reverse Sequence (5' - 3 ' )** |
| --- | --- | --- |
| **β-actin** | **AGTGTGACGTTGACATCCGT** | **GCAGCTCAGTAACAGTCCGC** |
| **IL12P40** | **CTGTGCCTTGGTAGCATCTATG** | **GCAGAGTCTCGCCATTATGATTC** |
| **MIP-1A** | **TTCTCTGTACCATGACACTCTGC** | **CGTGGAATCTTCCGGCTGTAG** |
| **IL10** | **CTTACTGACTGGCATGAGGATCA** | **GCAGCTCTAGGAGCATGTGG** |
| **IL6** | **TAGTCCTTCCTACCCCAATTTCC** | **TTGGTCCTTAGCCACTCCTTC** |
| **IFN**-**γ** | **TCAAGTGGCATAGATGTGGAAGAA** | **TGGCTCTGCAGGATTTTCATG** |
| **TNF**-**α** | **AGGCACTCCCCCAAAAGAT** | **CAGTAGACAGAAGAGCGTGGTG** |
| **IL1α** | **CGAAGACTACAGTTCTGCCATT** | **AAACTTCTGCCTGACGAGCTT** |
| **CB1** | **CTGATGTTCTGGATCGGAGTC** | **TCTGAGGTGTGAATGATGATGC** |
| **CB2** | **TGACAAATGACACCCAGTCTTCT** | **ACTGCTCAGGATCATGTACTCCTT** |
| **Occludin** | **TTCCTCTGACCTTGAGTGTGG** | **CTCTTGCCCTTTCCTGCTTT** |
| **Claudin-4** | **GTCCTGGGAATCTCCTTGGC** | **TCTGTGCCGTGACGATGTTG** |
| **ZO1/Tjp1** | **GCCGCTAAGAGCACAGCAA** | **GCCCTCCTTTTAACACATCAGA** |

**Abbreviation: IL, interleukin; MIP, macrophage inflammatory protein；TNF**-**α, tumour necrosis factor alpha; IFN**-**γ, Interferon γ; CB1, cannabinoid receptor 1; CB2, cannabinoid receptor 2; ZO1/Tjp 1, Zonula occludens-1/Tight junction protein1.**

**Supplementary Table S2. MRPP test used to analyze the β diversity in fecal analysis**

| **Group** | **A** | **observed-delta** | **expected-delta** | **Significance** |
| --- | --- | --- | --- | --- |
| **CP-L5** | **0.2173** | **0.4755** | **0.6076** | **0.001** |
| **DP-L5** | **0.0870** | **0.5276** | **0.5778** | **0.003** |
| **CP-DP** | **0.2134** | **0.4437** | **0.5641** | **0.001** |

**MRPP: Multi ResponsePermutation Procedure; Observe Delta: intragroup difference; Expect delta: intergroup difference; A>0: intergroup difference>intragroup difference; A<0: intragroup difference>intergroup difference; Significance<0.05: significant intergroup different.**


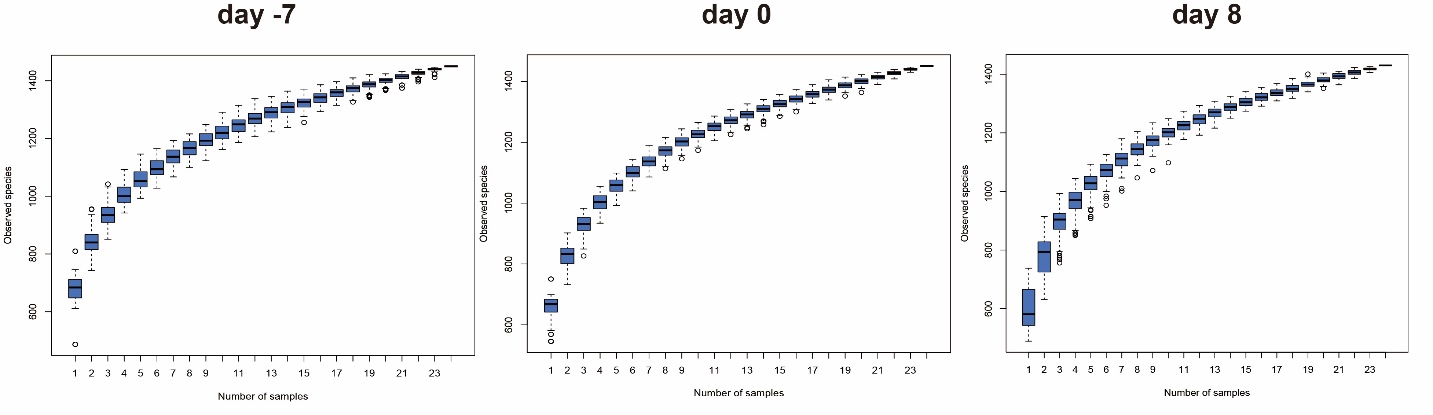


Figure S1 Species accumulation boxplot depicting the species richness in the three groups on days -7, 0 and 8.


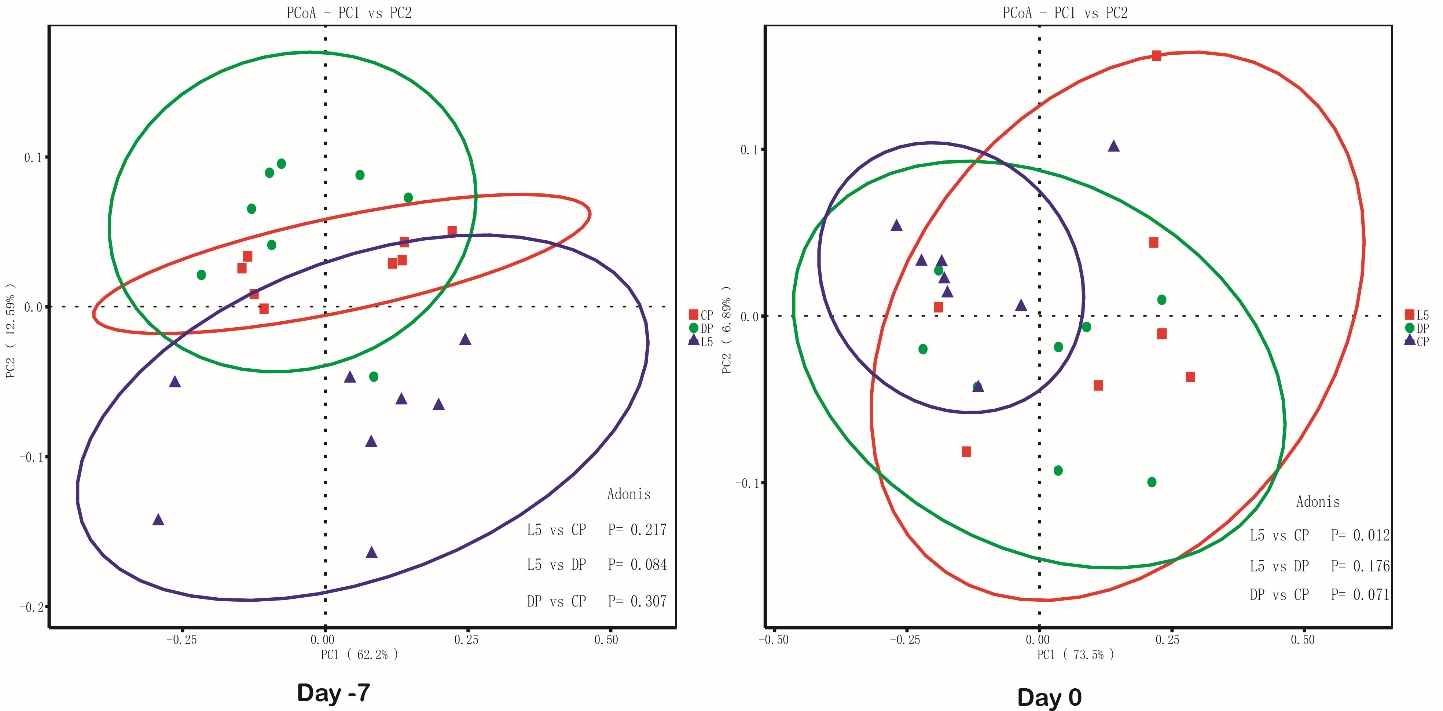
Figure S2 PCoA plot based on the weighted UniFrac distances among three groups on day -7 (left panel) and day 0 (right panel). Each point represents a sample. Adonis was used to test for microbial community clustering using weighted UniFrac distance matrices.


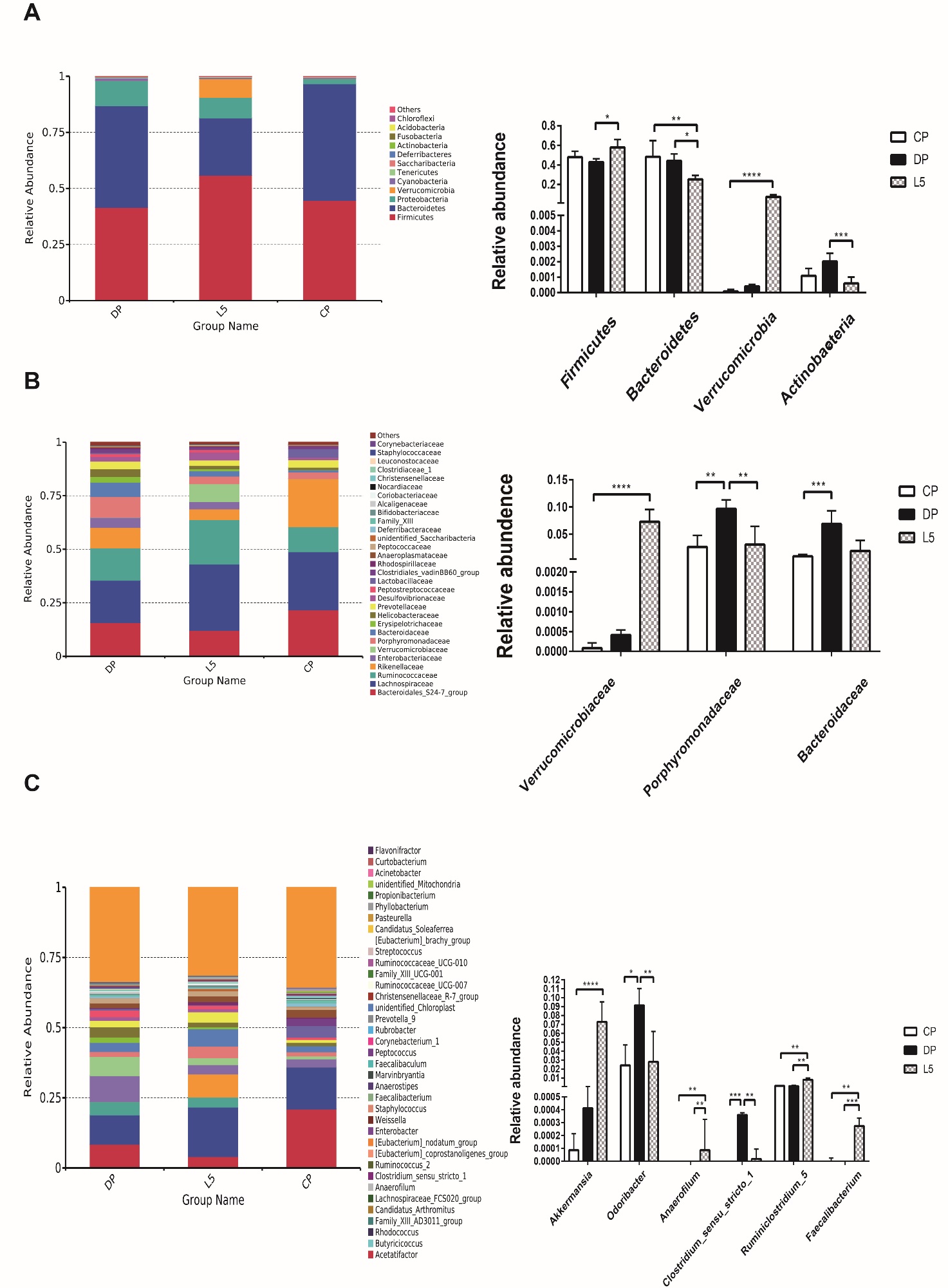


Figure S3 Relative abundance of taxa at the phylum (A), family (B) and genus (C) levels. Bar charts present the differences in the abundance of specific taxa among the three groups (day 8). All data are presented as medians with interquartile ranges. *P < 0.05, **P < 0.01, ***P < 0.001, and ****P < 0.0001 according to the Kruskal-Wallis tests.


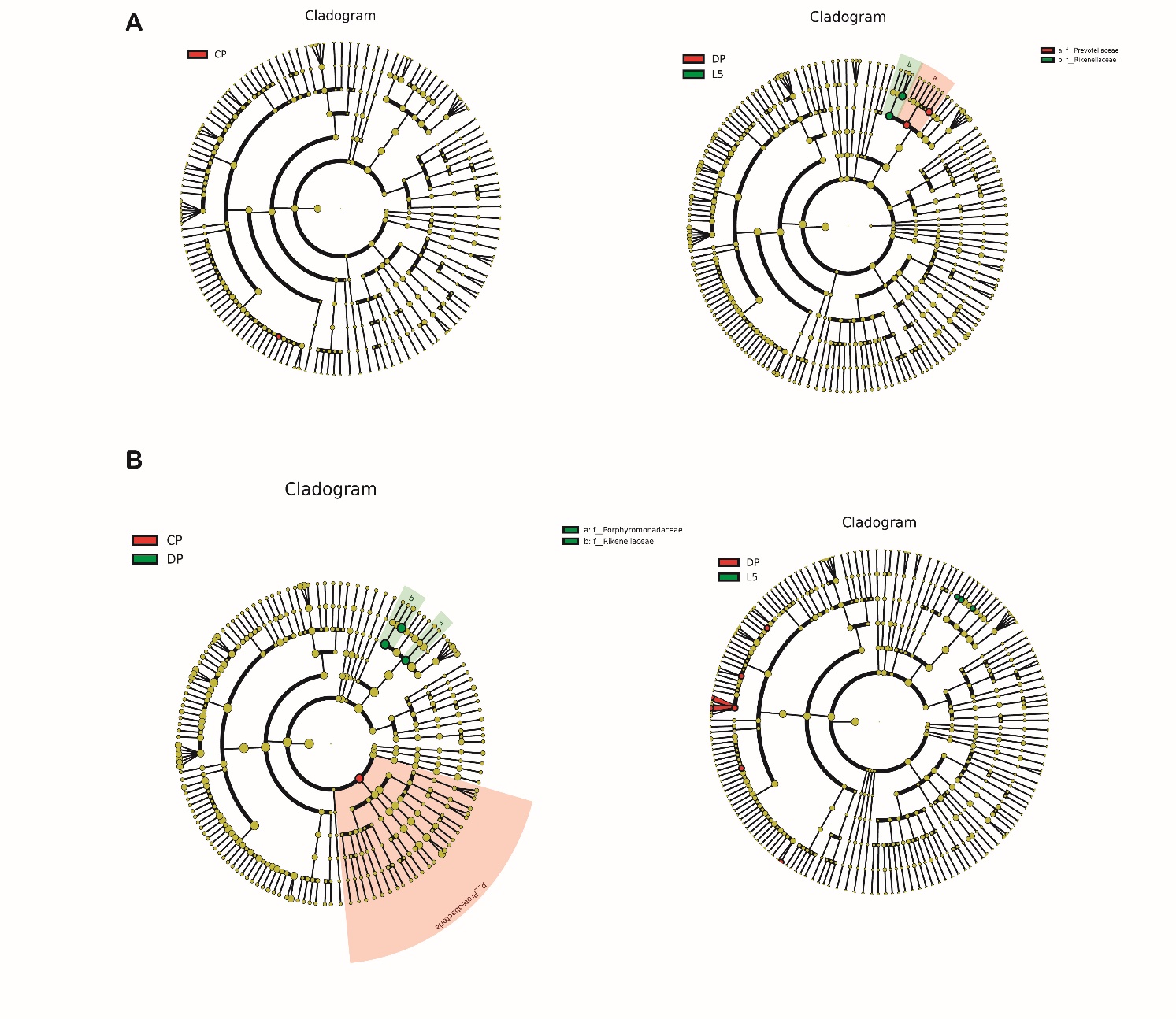


Figure S4 LEfSe analysis comparing differences between three groups on day -7 (A) and day 0 (B).


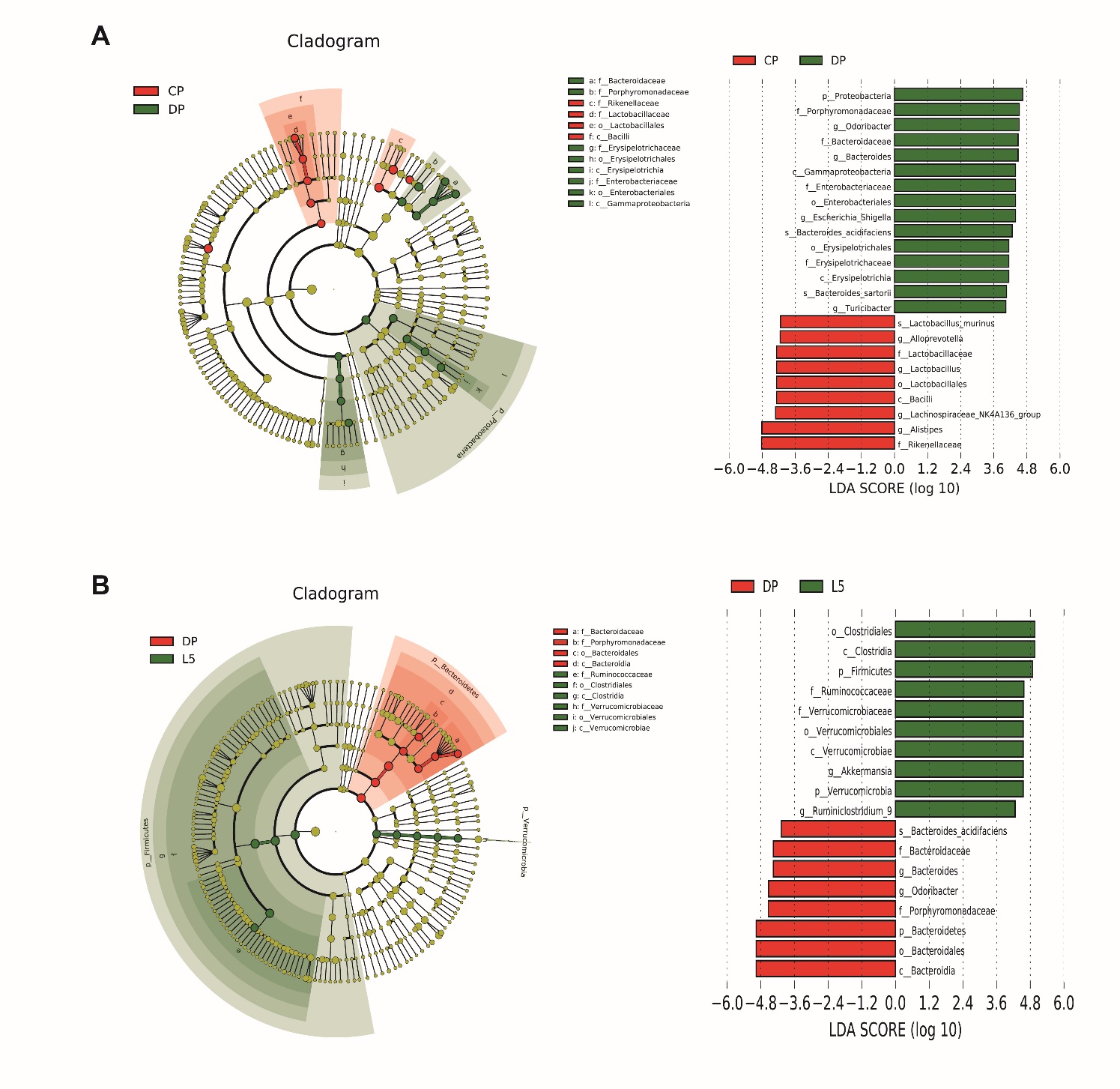


Figure S5 The *P. pentosaceus* LI05 treatment altered the gut microbial composition. (A) The LEfSe cladogram represents taxa enriched in the CP (red) and DP (green) groups (left panel) and discriminative biomarkers with an LDA score >4.8 (left panel) between two groups (day 8). (B) The LEfSe cladogram represents taxa enriched in the DP (red) and L5 (green) groups (left panel) and discriminative biomarkers with an LDA score >4.8 (left panel) between two groups.


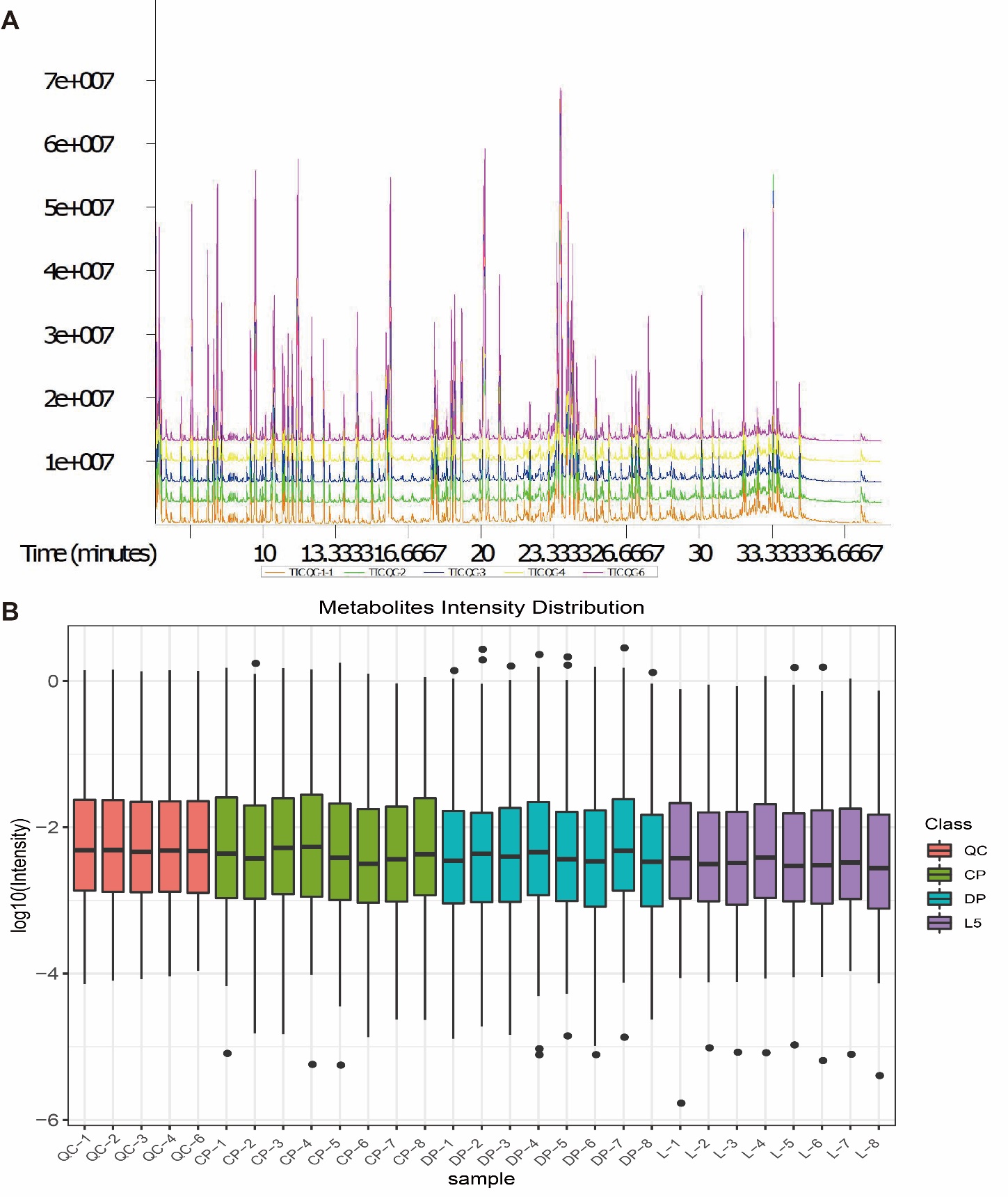


Figure S6 (A) The total ion stacking chromatogram of QC samples showed the small variation caused by instrument error. (B) The boxplot of the metabolite intensity distribution.


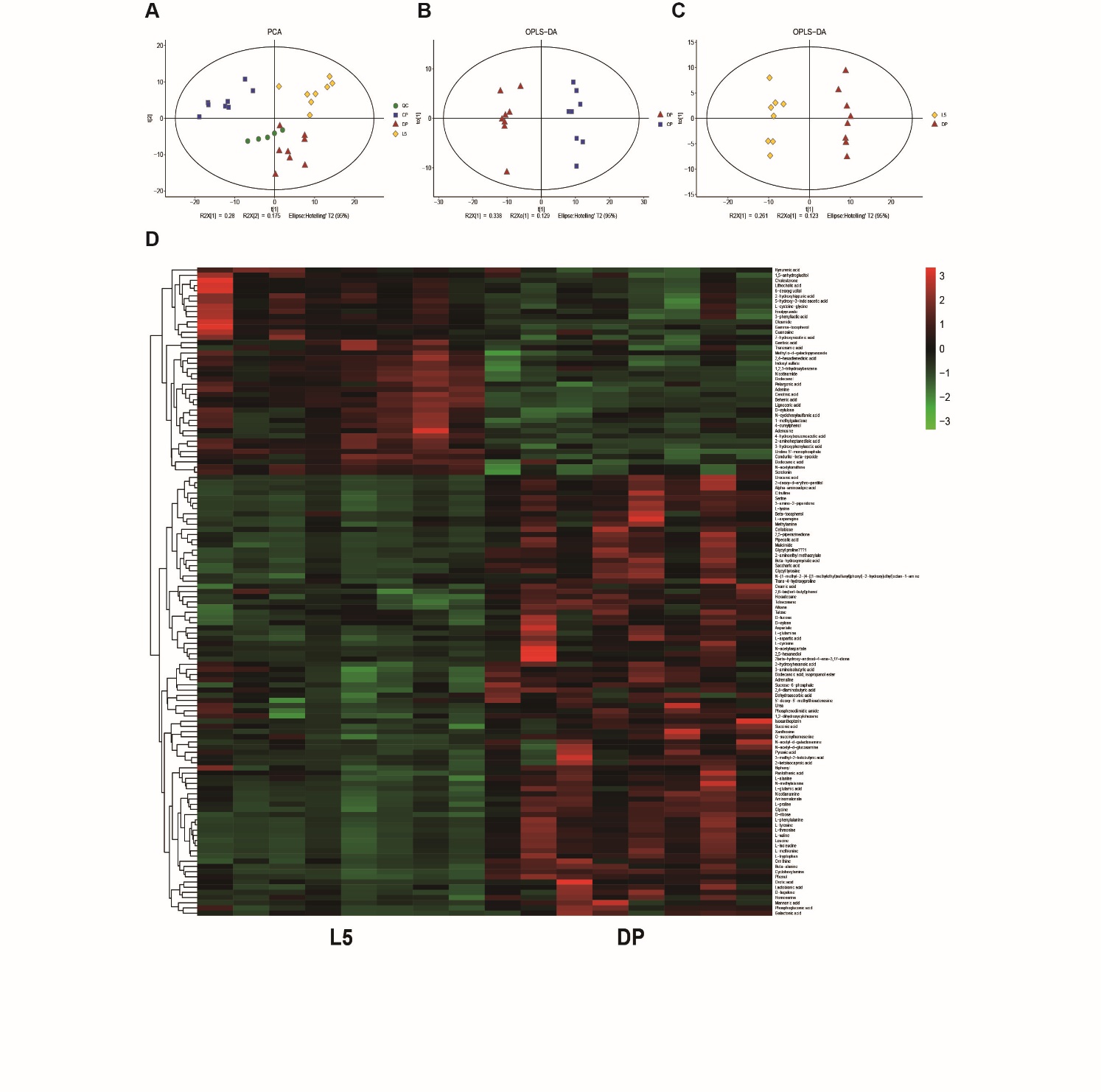


Figure S7 The *P. pentosaceus* LI05 treatment altered DSS-induced fecal metabolic profiles. (A) PCA plot comparing the QC (green), CP (blue), DP (red), L5 (yellow) groups. (B) OPLS-DA score plot comparing the CP (blue) and DP (red) groups. (C) OPLS-DA score plot comparing the L5 (yellow) and DP (red) groups. (D) Heat map showing the distribution of different levels of differentially altered metabolites between the L5 and DP groups based on the hierarchical clustering analysis. In the heat map profiles, relative values normalized to 3 and -3 are represented by different colors. Red indicates the high levels of differentially altered metabolites and green indicates low levels of differentially altered metabolites.


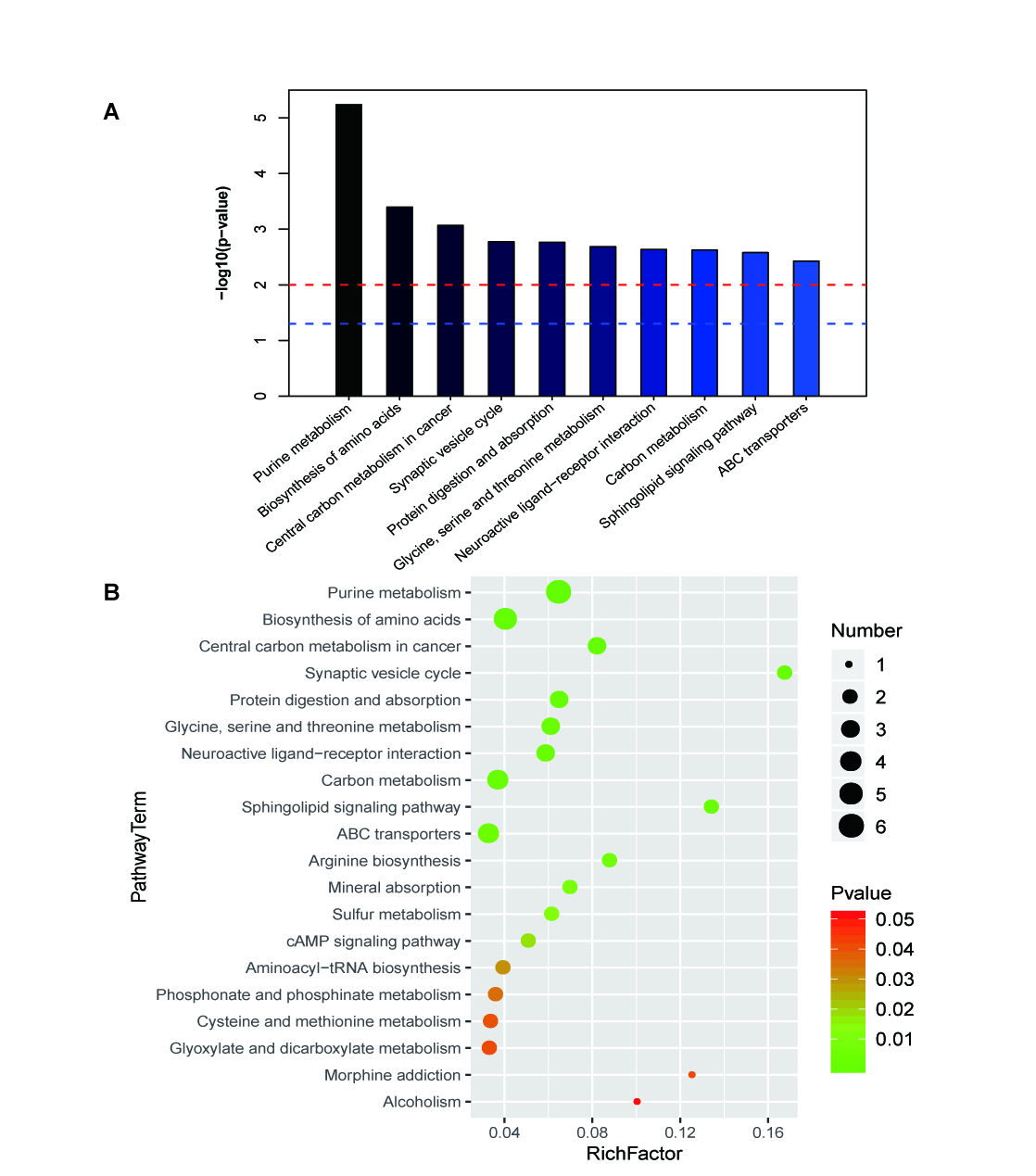


Figure S8 (A) Map of the top 10 metabolic pathways enriched in the L5 and DP groups. The red line indicates a P value=0.01, the blue line indicates a P value=0.05. (B) Bubble diagram of the top 20 metabolic pathways enriched in the L5 and DP groups. Enrichment factor = number of the significantly different metabolites/total metabolites in the pathway. The color change from red to green indicates a decrease in the P value. A larger bubble indicates a greater number of metabolites that were enriched in the pathway.
